# Comparative analysis of the *Spirulina platensis *subcellular proteome in response to low- and high-temperature stresses: uncovering cross-talk of signaling components

**DOI:** 10.1186/1477-5956-9-39

**Published:** 2011-07-15

**Authors:** Pavinee Kurdrid, Jittisak Senachak, Matura Sirijuntarut, Rayakorn Yutthanasirikul, Phuttawadee Phuengcharoen, Wattana Jeamton, Sittiruk Roytrakul, Supapon Cheevadhanarak, Apiradee Hongsthong

**Affiliations:** 1 BEC Unit, National Center for Genetic Engineering and Biotechnology, 49 Soi Theintalay 25, Thakham, Bangkhuntien, Bangkok, 10150, Thailand; 2 School of Bioresources and Technology, King Mongkut's University of Technology Thonburi, 49 Soi Theintalay 25, Thakham, Bangkhuntien, Bangkok, 10150, Thailand; 3 Pilot Plant Development and Training Institute, King Mongkut's University of Technology Thonburi, 49 Soi Theintalay 25, Thakham, Bangkhuntien, Bangkok, 10150, Thailand

## Abstract

The present study focused on comparative proteome analyses of low- and high-temperature stresses and potential protein-protein interaction networks, constructed by using a bioinformatics approach, in response to both stress conditions.

The data revealed two important points: first, the results indicate that low-temperature stress is tightly linked with oxidative stress as well as photosynthesis; however, no specific mechanism is revealed in the case of the high-temperature stress response. Second, temperature stress was revealed to be linked with nitrogen and ammonia assimilation. Moreover, the data also highlighted the cross-talk of signaling pathways. Some of the detected signaling proteins, e.g., Hik14, Hik26 and Hik28, have potential interactions with differentially expressed proteins identified in both temperature stress conditions. Some differentially expressed proteins found in the *Spirulina *protein-protein interaction network were also examined for their physical interactions by a yeast two hybrid system (Y2H). The Y2H results obtained in this study suggests that the potential PPI network gives quite reliable potential interactions for *Spirulina*. Therefore, the bioinformatics approach employed in this study helps in the analysis of phenomena where proteome analyses of knockout mutants have not been carried out to directly examine for specificity or cross-talk of signaling components.

## Introduction

Under thermal stress conditions, cells undergo many cellular modifications in order to survive and grow. These modifications are generated by a network of genes that are up- or down-regulated either simultaneously or in cascade. Among the well-known mechanisms related to temperature stress, maintenance of the homeoviscous adaptation of the cell membrane in order to retain the proper membrane flexibility is well defined.

*Spirulina *cells encounter temperature fluctuations, associated with outdoor mass cultivation, that have a relevant effect on biomass yield and the biochemical content of the cells. Some components with pharmaceutical benefits, such as unsaturated fatty acids in membrane lipids, have been shown to play vital roles in the response to temperature change. In addition, an association between fatty acid desaturation and temperature stress has been well established [1].

Physiological changes to the fatty acid content of the cytoplasmic membrane are also observed in *Spirulina platensis*. In comparison with cells maintained at the ideal growth temperature (35°C), *S. platensis *cells synthesize up to 23% more γ-linolenic acid (GLA; C18:3^Δ9,12,6^) after a temperature downshift (22°C), whereas the GLA level decreases approximately 30% upon a temperature increase (40°C) [2]. Therefore, extensive studies on the expression and regulation of desaturase genes in response to temperature changes have been performed by using a gene-by-gene approach [3].

High-throughput approaches such as proteomics have also been applied to analyze the temperature response of *Spirulina*. The proteomic analyses of the low- and high-temperature responses in *Spirulina *were performed by our research group using two-dimensional differential gel electrophoresis (2D-DIGE) coupled with protein identification by mass spectrometry [4]. As a result of the analysis of differential expression, four groups of proteins were found to be in common between *Spirulina *cells under both temperature stress conditions: (i) signal transduction proteins, (ii) chaperones, (iii) stress-related proteins and (iv) proteins related to DNA modification & repair [4,5]. Moreover, two groups of proteins, classified as channeling & secretion and photosynthesis, were detected as differentially expressed proteins only under low-temperature stress [4]. It is obvious that stress response mechanisms, when considered in isolation from other stresses in order to simplify interpretation, might not be comprehensive. Thus, it should be beneficial to understand a large part of the temperature stress response of *Spirulina *because temperature is an important environmental factor for the fatty acid contents in the cells, as mentioned above.

However, one limitation of the gel-based technique is that some differentially expressed proteins, such as those with an extreme pI or extreme molecular weight, cannot be detected. Thus, an aim of the present study was to perform a proteomic analysis of *Spirulina *in response to temperature reduction using iTRAQ-multidimensional liquid chromatography and tandem mass spectrometry. Moreover, the focus of the study was on comparative proteome analyses of low- and high-temperature stresses, as well as potential protein-protein interaction networks, constructed by using a bioinformatics approach, that respond to both stress conditions.

## Materials and methods

### Culture Conditions and Low-Temperature Exposure

**Figure 1:**
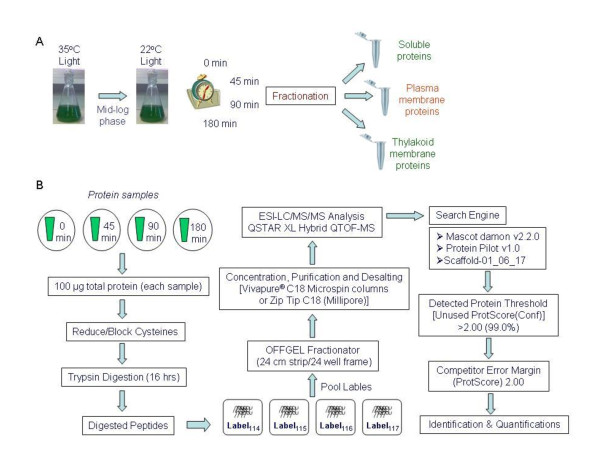
**Experimental design and workflow for the quantitative analysis of *Spirulina *in response to immediate temperature reduction**. (A) Experimental design to study the response of *Spirulina *to low-temperature stress represented by differentially expressed proteins in three subcellular fractions. (B) An illustration of the quantitative proteomic iTRAQ (4-plex)-multidimensional liquid chromatography-tandem mass spectrometry workflow.

Cultures of axenic *S. platensis*, strain C1, were grown at their optimal temperature, 35°C, under illumination by 100 μEm^-2 ^s^-1 ^fluorescent light with continuous stirring in 2 L of Zarrouk's medium [6]. For the low-temperature exposure, the culture was grown until the optical density at 560 nm reached 0.4 (mid-log phase), at which time a cell sample was harvested by filtration before shifting the growth temperature (t = 0 min). The growth temperature was immediately shifted from 35°C to 22°C, and the culture was incubated for 45, 90 or 180 min before cell harvesting (Figure 1a). It should be noted that the experiment was carried out in the same manner for high temperature (40°C) exposure [5]. However, in the present study, only the low temperature exposure samples were subjected to the iTRAQ-multidimensional liquid chromatography and tandem mass spectrometry.

### Preparation of Subcellular Fractions, Protein Extraction and Protein Quantification

The harvested cells were washed and then lysed using a French press at 700 psi, and the soluble protein fraction was collected. The plasma and thylakoid membranes were isolated on a sucrose gradient as described by Murata and Omata [7]. The washed membrane pellet was resuspended in the dissolving buffer containing 2 M thiourea, 8 M urea, 20 mM Tris, 30 mM DTT, 1% (v/v) IPG buffer, 0.05% (w/v) β-dodecyl maltoside, and 4% (w/v) CHAPS [4]. For each of the time periods for each of the subcellular fractions, 100 μg of protein was precipitated in a 1:6 volume of acetone overnight at -20°C and resuspended in the appropriate buffers for iTRAQ labeling.

### Four-Plex Isobaric Tag Peptide (iTRAQ) Labeling

Protein concentration of each sample was measured before subjected to iTRAQ labeling. For each of the time periods for each of the subcellular fractions, 100 μg of protein was reduced and alkylated as described by the manufacturer's protocol (Applied Biosystems, USA). Subsequently, the protein samples were digested with trypsin (Promega, USA) (1:5) overnight at 37°C. Peptide samples from the four time periods (0, 45, 90 and 180 min) were then labeled with 4-plex iTRAQ (114, 115, 116 and 117, respectively) using an iTRAQ labeling kit (Applied Biosystems) according to the manufacturer's protocol. The subcellular fractions from all time periods were combined, vacuum evaporated and stored at -20°C prior to separation by liquid chromatography.

### Liquid Chromatography and Tandem Mass Spectrometry

The workflow of the experiment is shown in Figure 1b. Off-gel fractionation (Agilent, USA) with a buffer of pH 3-10 was performed to increase the sensitivity of the protein analysis, according to the manufacturer's protocol. After the twenty-four off-gel fractions were cleaned using ZipTips^® ^(Millipore, USA), the samples were subjected to liquid chromatography-tandem mass spectrometry (LC-MS/MS) using a Famos-Switchos-Ultimate nano-LC system (Dionex, LC packing, The Netherlands) interfaced with a QSTAR XL (Applied Biosystems; MDS-Sciex) tandem ESI-QUAD-TOF MS. Concentrated off-gel fractions were resuspended in 0.1% formic acid, injected and captured in a 0.3 × 5 mm C18 trapping cartridge (LC Packings). The trapped peptides were eluted onto a 0.075 × 100 mm C18 analytical column (packed in-house with 5-μm particle size packing material from Column Engineering, USA), using an automated binary gradient with a flow of 100 nl/min across a 0% to 90% acetonitrile gradient in 0.1% formic acid over a 110-min period. The eluted peptides were separated through the column and then analyzed online by tandem mass spectrometry.

Eluted peptides from the LC column were sprayed directly into the orifice of the mass spectrometer, which was run in IDA (information dependent acquisition) mode, selecting all 2+ to 3+ charged ions with a signal intensity greater than eight counts per second over the specified mass range. For experimental run 1, all fractions were run and scanned in the m/z range of 300-1800 amu. For run 2, all fractions were run and scanned in the m/z range of 100-1800 amu, with an exclusion list derived from the previously identified peptides. Further details on the MS/MS conditions are described elsewhere [8]. Three subcellular fractions obtained from three independent experiments (low-temperature stress) were analyzed.

### Proteomic Data Analysis

The quantification analysis of iTRAQ-peptide spectra was performed using ProteinPilot™ software v. 1.0 (Applied Biosystems) and Scaffold software v. 1.7 (Proteome Software, Inc.). However, the *S. platensis *strain C1 genome database was not available in the database connected to ProteinPilot™ and Scaffold software. Result files of the three subcellular fractions obtained by using ProteinPilot™ were shown as Additional file 1, 2, 3 Table S1a, S1b, S1c. Thus, protein identifications were carried out further by converting the raw data files (*.wiff) to *.mzXML and then to *.mgf files, consecutively, followed by a search against an in-house *S. platensis *C1 protein database (6360 ORFs, accessed March 2009, unpublished database) using the Mascot Search algorithm. However, the open reading frame (orf) code of the published genome of *Synechocystis *that matched the designated *Spirulina*-orf was also reported. The quantification and identification data were integrated using Microsoft Access. The significant differentially expressed proteins were filtered by the same criteria as that of the 2D-DIGE experiment [4,5], *p*-value ≤ 0.05 and fold change (log scale) ≥ 1.5 (for up-regulated proteins) or ≤ -1.5 (for down-regulated proteins). Moreover, the criteria for protein ID was sequence similarity by matching peptide sequences of each identified protein obtained from LC-MS/MS coupled with ProteinPilot and Scaffold with *S. platensis *strain C1 protein sequence database, translated from its genome sequence.

### Transcriptional Analysis by RT-PCR

*Spirulina *RNA isolation was performed as described previously [9]. The expression levels of the gene transcripts of interest were analyzed by RT-PCR. RT-PCR kits were selected according to the size of the gene transcripts. The AccessQuick™ RT-PCR System (Promega) and the SuperScript III First-Strand Synthesis System coupled with the Platinum ^® ^Taq DNA Polymerase High Fidelity kit (Invitrogen, USA) were used for gene sizes ranging from 500 bp - 2 kb and 2 kb - 6 kb, respectively, according to the manufacturers' protocols. Details on the primers are shown in Additional file 4 Table S2. Quantification of the RT-PCR products was performed by measuring the densities of the bands using the Image Quant TL program (GE Healthcare Biosciences). Normalization of the RT-PCR product levels was then carried out by comparing the density of the designated band to the density of the 16S rRNA bands.

### Clustering of Protein Expression Patterns

The protein expression dataset (obtained by using LC-MS/MS technique) was validated for the input well-form of protein ratio values. Then, the null values and ratios that were extremely high or low, relative to the threshold value of 1e+-10, were filtered out. K-mean clustering was applied to obtain three major temporal response patterns, resistance (short-term response only), adaptation (long-term response only) and sustained tolerance (short- and long-term response) patterns. Other proteins, for which expression patterns cannot be clustered into the three majority groups, were clustered separately. A good k-profile number was chosen by simulation according to Martin et al. [10].

### Potential Protein-Protein Interaction (PPI) Network Construction

A protein-protein interaction (PPI) network in *Spirulina *was constructed on the prototype PPI database of *Synechocystis *[11]. Prototype construction was based on a graph in which nodes and edges represent proteins and interactions, respectively. Each interaction was experimentally identified by a yeast two-hybrid system. An edge was drawn from a bait protein and targeted to its prey protein at the head of the edge. Homologous proteins identified by BLAST similarity searches with significance values less than 1 × 10^-10 ^were mapped to their best-hit *Synechocystis *protein nodes. The connectivity of the overall network was analyzed for maximal connected subgraphs using the concepts of weakly connected components and biconnected components. In each sub-network (connected components), a protein in the most highly connected part was selected, along with the drawn interactions that target its reachable proteins. Those interactions can be seen as a bridge between proteins, which in graph theory are classified as cut vertices. Finally, differentially expressed proteins in *Spirulina *were mapped to their corresponding nodes. The height of each node represents the level of differential expression.

### Protein-Protein Interaction Analysis by Yeast Two Hybrid System (Y2H)

Y2HGold and Y187 (Clontech, USA) were used for designated plasmid DNA transformation. Testing for bait autoactivation, which leads to false positives, is necessary prior to the experiments. The bait-containing plasmid, Y2HGold, and the prey-containing plasmid, Y187, were mated in 300 μl of 2XYPDA broth. In addition, the positive control (plasmid pGBKT7-p53 in Y2HGold mated with plasmid pGADT7-T in Y187) and negative control (plasmid pGBKT7-Lam in Y2HGold mated with plasmid pGADT7-T in Y187) were mated under identical conditions. The yeast mating cultures were incubated at 30°C, with shaking at 200 rpm for 24 hr. Then, the cultures were spread onto SD/-Leu/-Trp (DDO) and SD/-Leu/-Trp/X-α-gal/AbA dropout (DDO/X/A) plates, and incubated at 30°C for 3 days. Subsequently, the blue colonies were picked and streaked onto high stringency SD/-Ade/-His/-Leu/-Trp/X-α-gal/AbA dropout (QDO/X/A) plates, followed by incubation at 30°C for 3-5 days. Furthermore, the switching of yeast strains for the bait-containing plasmid and the prey-containing plasmid were done to confirm the specific interaction between bait and prey proteins. Positive results were expected for the specific interaction.

## Results

### General overview

Proteomic analyses of *S. platensis *responding to immediate low- and high-temperature shifts, from 35°C to 22°C and 40°C, respectively, were performed by using two approaches, 2D-DIGE (the previous studies [4,5]) and iTRAQ LC-MS/MS (the present study). The levels of protein expression after 180 min of temperature shift were compared to those of the control cyanobacteria grown at 35°C (0 min). In the present study, 52 proteins were identified as differentially expressed proteins in response to the temperature reduction. Twenty-eight, sixteen and two differentially expressed proteins were revealed in the soluble, thylakoid membrane (TM) and plasma membrane (PM) fractions, respectively, whereas, two proteins were detected in all subcellular fractions, and three proteins were found in soluble and TM fractions [Additional file 5 Table S3]. When the differentially expressed proteins identified by 2 different proteomic techniques, 2D-DIGE (the previous studies [4,5]) and iTRAQ LC-MS/MS (the present study), were compared, only one protein, GroEL, detected by both techniques was identified; furthermore, 142 and 51 proteins were uniquely detected by 2D-DIGE and LC-MS/MS, respectively.

When the two stress conditions were compared, a set of 100 proteins, including 3 signaling proteins, was identified as a response to the low-temperature shift, and a set of 69 proteins, including 17 signaling proteins, was identified as a response to the high-temperature shift. Furthermore, a set of 20 proteins, including 3 signaling proteins, was found to be common between the two temperatures [Additional file 6, 7, 8 Table S4a, S4b, S4c].

The three sets of proteins mentioned above were mapped onto a Cyanobase protein-protein interaction (PPI) network to construct a *Spirulina *potential PPI network [Additional file 9, 10, 11, 12, 13, 14, 15, 16, 17 Figure S1a, S1b, S1c, S1d, S1e, S1f, S1g, S1h, S1i], and potential interactions were tested by using a yeast two hybrid system. Moreover, some proteins of interest were analyzed at the transcriptional level in order to study their regulation [Additional file 18 Figure S2].

### Clustering of protein expression patterns

**Figure 2:**
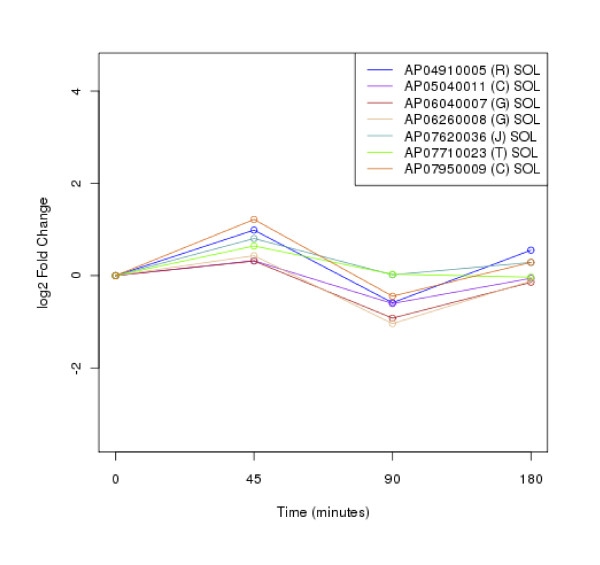
**Cluster 1 of expression pattern clustering of the differentially expressed proteins detected by using LC-MS/MS technique after the low-temperature exposure**.

**Figure 3:**
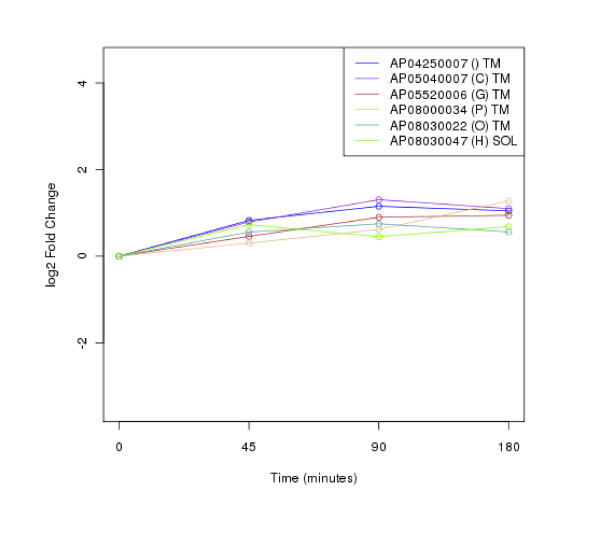
**Cluster 2 of expression pattern clustering of the differentially expressed proteins detected by using LC-MS/MS technique after the low-temperature exposure**.

**Figure 4:**
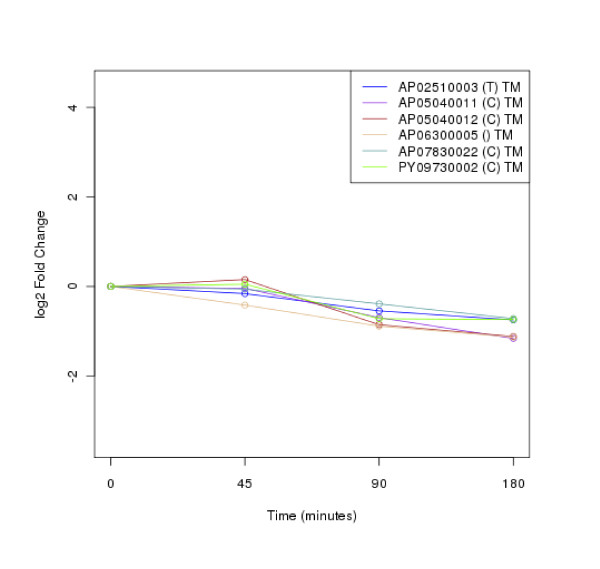
**Cluster 3 of expression pattern clustering of the differentially expressed proteins detected by using LC-MS/MS technique after the low-temperature exposure**.

**Figure 5:**
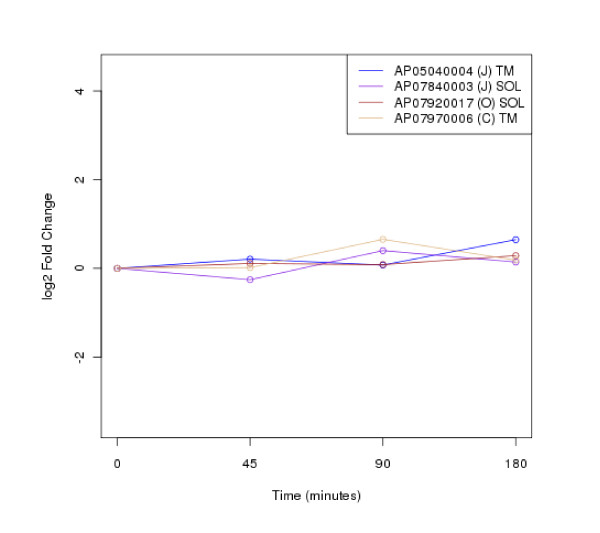
**Cluster 4 of expression pattern clustering of the differentially expressed proteins detected by using LC-MS/MS technique after the low-temperature exposure**.

**Figure 6:**
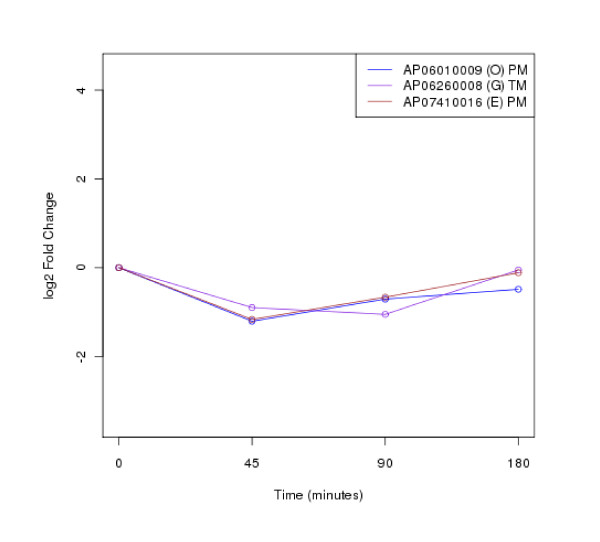
**Cluster 5 of expression pattern clustering of the differentially expressed proteins detected by using LC-MS/MS technique after the low-temperature exposure**.

**Figure 7:**
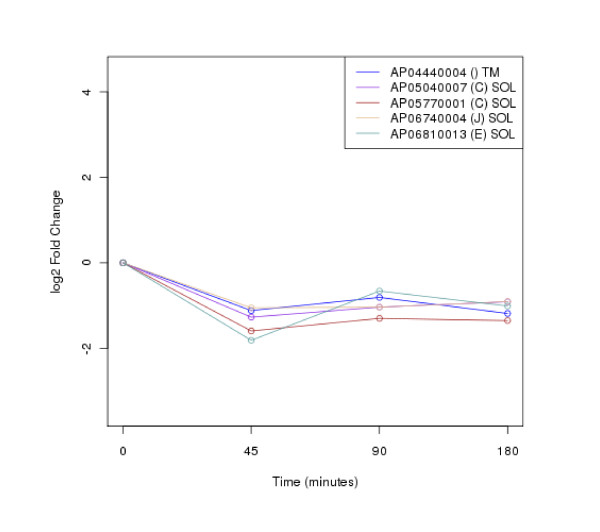
**Cluster 6 of expression pattern clustering of the differentially expressed proteins detected by using LC-MS/MS technique after the low-temperature exposure**.

**Figure 8:**
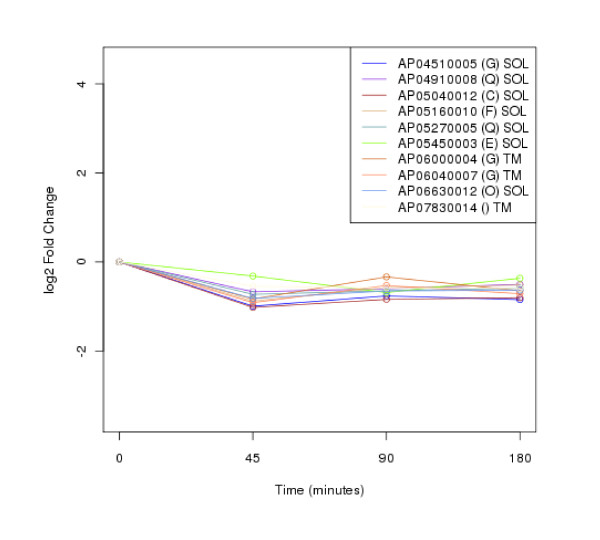
**Cluster 7 of expression pattern clustering of the differentially expressed proteins detected by using LC-MS/MS technique after the low-temperature exposure**.

**Figure 9:**
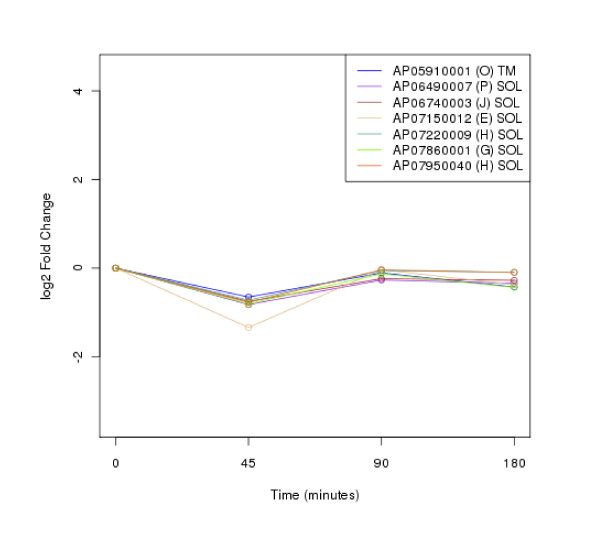
**Cluster 8 of expression pattern clustering of the differentially expressed proteins detected by using LC-MS/MS technique after the low-temperature exposure**.

**Figure 10:**
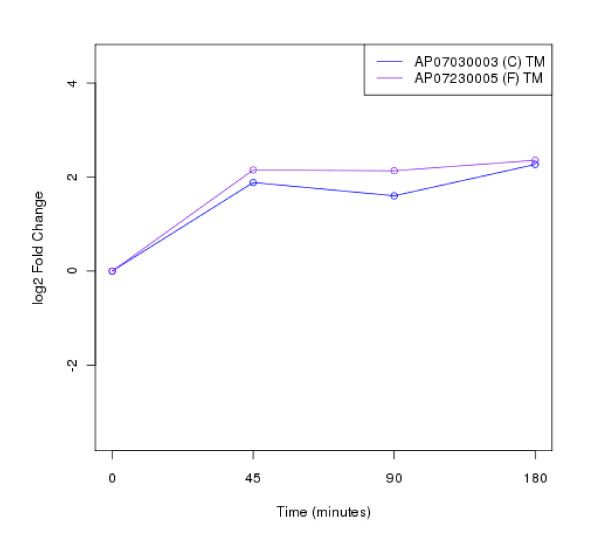
**Cluster 9 of expression pattern clustering of the differentially expressed proteins detected by using LC-MS/MS technique after the low-temperature exposure**.

**Figure 11:**
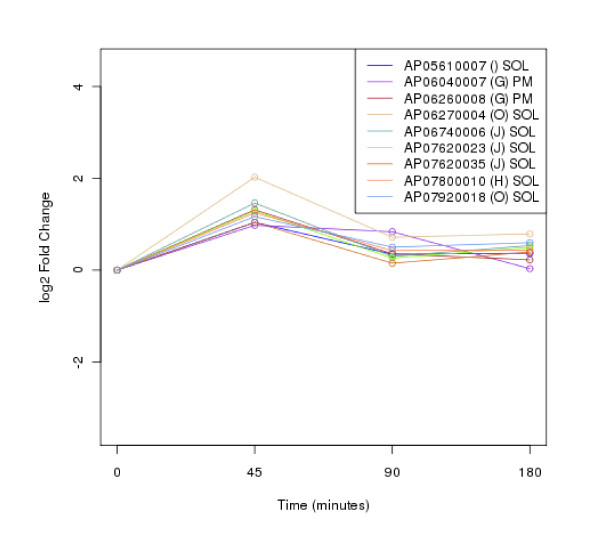
**Cluster 10 of expression pattern clustering of the differentially expressed proteins detected by using LC-MS/MS technique after the low-temperature exposure**.

The clustering of protein expression patterns in response to low-temperature stress was shown in Figure 2, 3, 4, 5, 6, 7, 8, 9, 10, 11 and Additional file 19 Table S5. The expression patterns were classified into 10 clusters. The summary of major protein expression patterns, resistant, adaptation and sustained tolerance, observed after exposure to the low-temperature stress by using LC-MS/MS technique was shown in Table 1.

**Table 1:** Summary of the major protein expression patterns observed after exposure to the low-temperature stress by using LC-MS/MS technique.

Expression pattern	Proteins in trend (%)	COG-function
Short-term only (Resistant)	27.1	C, G, H, J, O, R, T
Long-term only (Adaptation)	10.2	C
Short- and long-term (Sustained tolerance)	55.9	C, E, F, G, H, J, O, P, Q, R, T
Other	6.8	C, J, O

(**Note: **COG class; C: Energy production and conversion, E: Amino acid transport and metabolism, F: Nucleotide transport and metabolism, G: Carbohydrate transport and metabolism, H: Coenzyme transport and metabolism, J: Translation, ribosomal structure and biogenesis, O: Post-translational modification, protein turnover, chaperone functions, P: Inorganic ion transport and metabolism, Q: Secondary metabolites biosynthesis, transport and catabolism, R: General Functional Prediction only, T: Signal transduction mechanisms)

### Low-temperature responsive proteins

The three signaling proteins found only under the low-temperature stress condition are AP07580006, AP06700009 and AP07710023, which are matched with sll0821, sll5060 (or Hik14) and slr0947 of *Synechocystis*, respectively [Additional file 6 Table S4a].

Besides the signaling proteins, there are 97 proteins that responded to the low-temperature condition, as detected by 2D-DIGE and LC-MS/MS approaches [Additional file 6 Table S4a]. In addition to ATP synthase, chaperones, ribosomal proteins and proteins associated with post-translational modification, most of the cold-responsive proteins are involved in oxidative stress, nitrogen & ammonia assimilation and photosynthesis. A well-known oxidative stress-related protein, peroxidase, was up-regulated after the low-temperature exposure; however, other proteins involved in oxidative stress, such as (p)ppGpp synthetase, argininosuccinate synthase and glutamine synthetase, were down-regulated.

In the case of proteins related to nitrogen and ammonia assimilation, NADH dehydrogenase (ndh), nitrate ABC transporter (NrtD) and PEP carboxylase (Ppc) were differentially expressed. The ndh was up-regulated, whereas the other two were down-regulated [Additional file 6 Table S4a].

Moreover, several photosynthetic proteins were differentially expressed in response to the stress, including uroporphyrinogen decarboxylase (UroD or HemE), magnesium chelatase (ChlI), photochlorophyllide reductase (ChlN), PsaB, PsaF from photosystem I (PSI), PSI assembly protein (Ycf4), Rubisco small subunit (RbcS) and a Rubisco related protein, CcmM [Additional file 6 Table S4a] [4]. It should be noted that only PsaB was down-regulated, whereas others photosynthetic proteins were up-regulated upon immediate temperature reduction.

We examined the levels at which the expression of the signaling proteins (AP07580006 or Sll0821), HemE, Ppc and Hsp90 were regulated. The mRNA and protein levels of the signaling protein and HemE were correlated, indicating that the proteins are most likely regulated at the transcriptional level [Additional file 18 Figure S2] (Table 2). In contrast, the transcript levels of Ppc and Hsp90 did not correlate with their protein levels, suggesting that the proteins are possibly regulated at the post-transcriptional or post-translational level [Additional file 18 Figure S2] (Table 2).

**Table 2:** Regulation levels of several differentially expressed proteins of interest in response to temperature changes.

ORF	Protein name	Regulation level	Localization	Detected condition
AP05380002	DEAD/DEAH box helicase domain protein (membrane helicase)	Transcriptional level	PM, SOL, TM	Common
AP06740013	Ferredoxin-glutamate synthase	Transcriptional level	PM, SOL, TM	Common
AP06900013	Uroporphyrinogen decarboxylase	Transcriptional level	TM	Low-temperature
AP07580006	Response regulator (CheY like-GGDEF containing protein)	Transcriptional level	PM	Low-temperature
AP07670017	Two-component sensor histidine kinase	Transcriptional level	PM, SOL	Common
AP08030020	Heat shock protein 90 (Hsp90)	PTC or PTL or EA	TM	Low-temperature
AP08030057	Phosphoenolpyruvate carboxylase	PTC or PTL or EA	SOL	Low-temperature
AP08040017	Chaperone GroEL	Transcriptional level	SOL, TM	Common

*PTC, PTL and EA represent post-transcriptional level, post-translational level and enzymatic activity level, respectively.

**Note: **The figures that represent mRNA levels and protein levels of the proteins of interest shown in this table were Additional file 10 Figure S2. PM, SOL and TM represent plasma membrane, soluble, and thylakoid membrane fractions, respectively.

### High-temperature responsive proteins

In addition to our previous report [5], as many as seventeen signal transduction proteins were classified as heat-responsive proteins. Fourteen of them were up-regulated and three proteins were down-regulated [Additional file 7 Table S4b]. In addition to signaling and energy production related proteins, other groups of proteins found only after temperature elevation were (i) chaperone proteins, e.g., DnaK; (ii) translational proteins, e.g., Rps; (iii) transcriptional regulators, e.g., LysR and Baf; (iv) lipid desaturation proteins, e.g., Δ^9 ^desaturase; and (v) translocation systems, e.g., twin arginine translocation protein.

### Proteins in common between the two temperatures

The three signaling proteins found in common after the exposure to low-and high-temperature stresses are AP04840005, AP07350018 and AP07670017. The latter gene matched with sll0779 of *Synechocystis*, whereas the first two did not match with any *Synechocystis *gene [Additional file 8 Table S4c]. In addition to signal transduction proteins, six groups of proteins were identified as common between the two temperature stresses: (i) chaperones, e.g., ClpB and GroEL; (ii) proteins related to nitrogen and ammonia assimilation, e.g. NarB and ferredoxin-dependent glutamate synthase (GltB); (iii) stress-related proteins, e.g., DEAD/DEAH box helicase and S-adenosyl-L-homocysteine (SAH) hydrolase; (iv) post-translational modification, e.g., glycosyl transferase; (v) DNA damage and DNA repairing systems, e.g., exonuclease (SbcC); and (vi) transporters, e.g., ABC transporter [Additional file 8 Table S4c]. It is noteworthy that all the proteins found in common between the two stress conditions were up-regulated.

In case of transcriptional analysis, the transcriptional levels of the two component sensor histidine kinase (AP07670017 or sll0779), GroEL, GltB and DEAD/DEAH box helicase were studied by using RT-PCR. The results showed correlation between the transcript levels and the protein levels, indicating regulation was at the transcriptional level [Additional file 18 Figure S2] (Table 2).

### Potential protein-protein interactions

The three sets of proteins, low-temperature responsive proteins, high-temperature responsive proteins and proteins in common between the two temperatures, mentioned above were mapped onto a Cyanobase protein-protein interaction (PPI) network to construct a *Spirulina *potential PPI network [Additional file 9, 10, 11, 12, 13, 14, 15, 16, 17 Figure S1a, S1b, S1c, S1d, S1e, S1f, S1g, S1h, S1i].

### PPI analysis by using yeast two hybrid system (Y2H)

**Figure 12:**
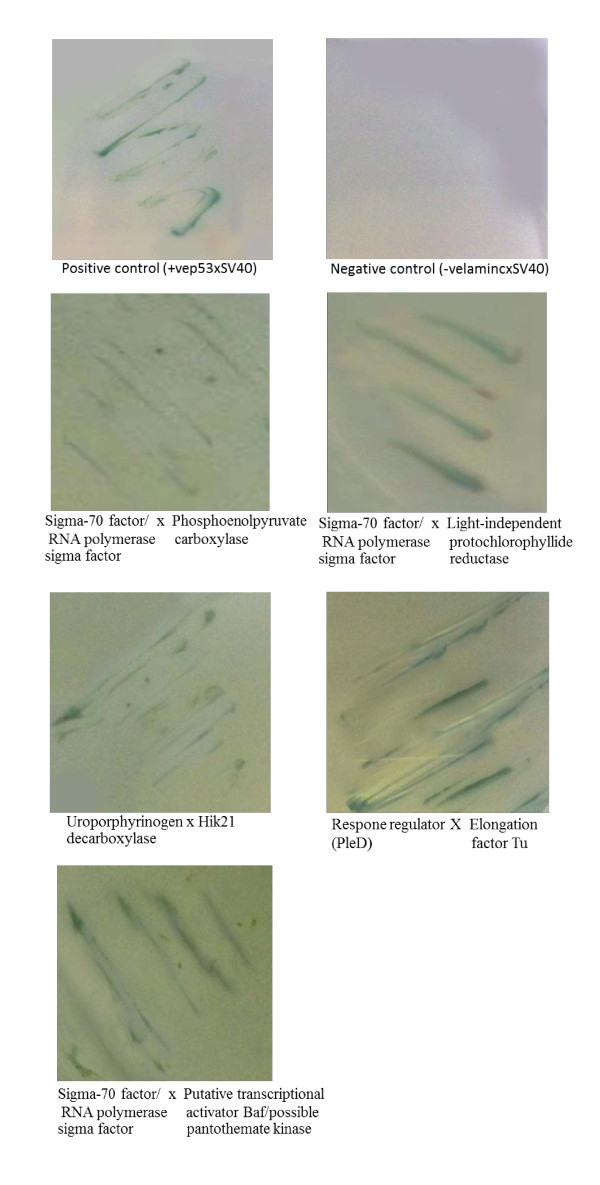
**Positive results of the designated protein-protein interactions using a yeast two hybrid system**.

Five pairs of potentially interacting proteins obtained from the potential PPI network were tested for their interactions by using a Y2H system (Figure 12). The results clearly showed that SigA interacts with ChlN, Ppc and Baf, whereas the cut-vertex two component signaling system (Hik21 or AP07430015) interacted with HemE or UroD. Moreover, the Y2H also gave a positive result for PleD (a response regulator/AP07540011) and EfTu.

## Discussion

### Clustering of protein expression patterns

The protein expression pattern clustering detected three clusters, representing resistance, adaptation and sustained tolerance proteins [5,12], in addition to the other pattern. If all differentially expressed proteins in response to low-temperature stress detected by using LC-MS/MS technique are set as 100%, the percentages of the resistance, adaptation and sustained tolerance groups are 27.1%, 10.2% and 55.9% respectively. The results shown in Figure 2, 3, 4, 5, 6, 7, 8, 9, 10, 11 and Table 1 demonstrate that the majority of proteins belong to the sustained tolerance expression pattern, which is similar to that of the high-temperature stress response reported earlier by our group [5].

It is noteworthy that the energy production and conversion related proteins in the group of ATPase and photosystem I & II detected in the thylakoid membrane fraction are the only members of the adaptation group (Table 1) [Additional file 19 Table S5].

### Low-temperature responsive proteins and their potential PPI

**Figure 13:**
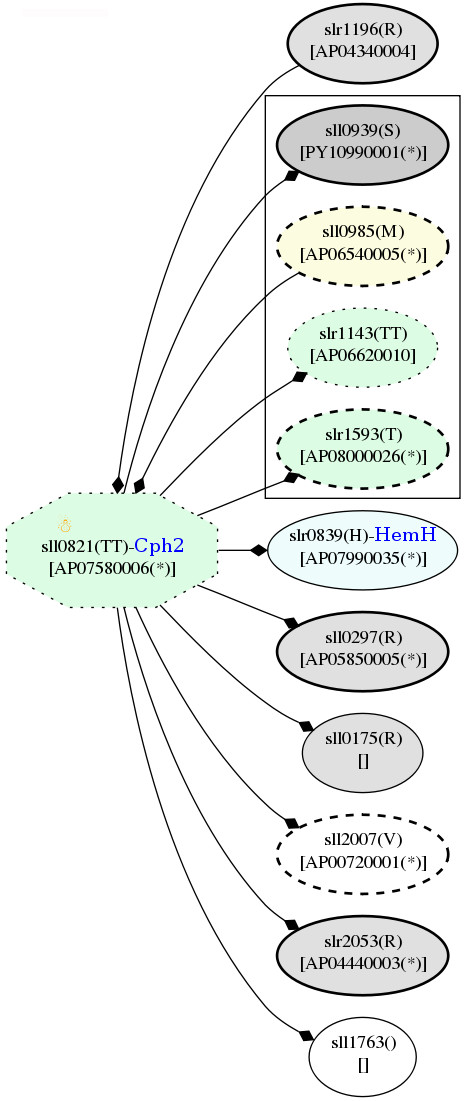
**Snapshots of AP07580006, two component response regulator, in the potential protein-protein interaction network in *Spirulina *in response to the temperature stresses**. The networks were constructed based on the data from Cyanobase.

**Figure 14:**
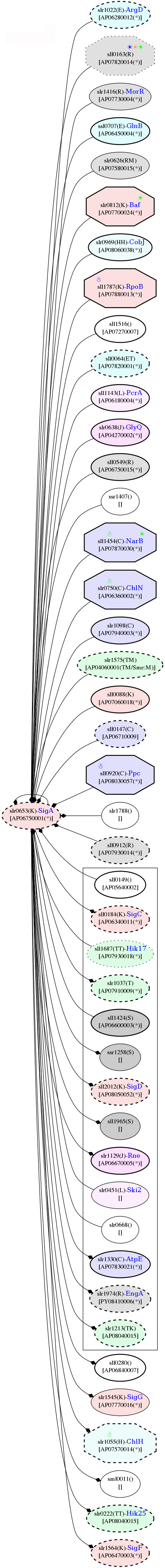
**Snapshots of AP06750001, SigA or RpoDI, in the potential protein-protein interaction network in *Spirulina *in response to the temperature stresses**. The networks were constructed based on the data from Cyanobase.

According to the potential PPI network, a low-temperature responsive signaling protein, AP07580006 interacts with several hypothetical proteins, ferrochelatase (HemH), and two response regulators that contain a CheY-like receiver and GGDEF domains (Figure 13). In contrast, no potential interaction was found for AP07710023 signaling proteins. However, AP06700009 or Hik14 associated with SigG, which interacted with RpoDI or SigA, the expression level of which was up-regulated about 1.46 fold (a bit less than the cut-off at 1.5 fold) under low-temperature stress [Additional file 9 Figure S1a]. Interestingly, SigA itself interacts with the photosynthetic protein ChlN, as well as proteins associated with nitrogen and ammonia assimilation: Ppc, nitrate reductase (NarB) (found as a common up-regulated protein under low- and high-temperature stress), a nitrate regulatory protein (GlnB) and a transcription regulator (Baf; up-regulated under temperature elevation) (Figure 14). It should be noted that GlnB was 1.36 fold significantly down-regulated (9% less than the cut-off level). Furthermore, it should be noted that SigA also associates with another signal protein, Hik25 (AP08040015), whose expression level did not vary after exposure to the temperature stress [Additional file 9 Figure S1a].

**Figure 15:**
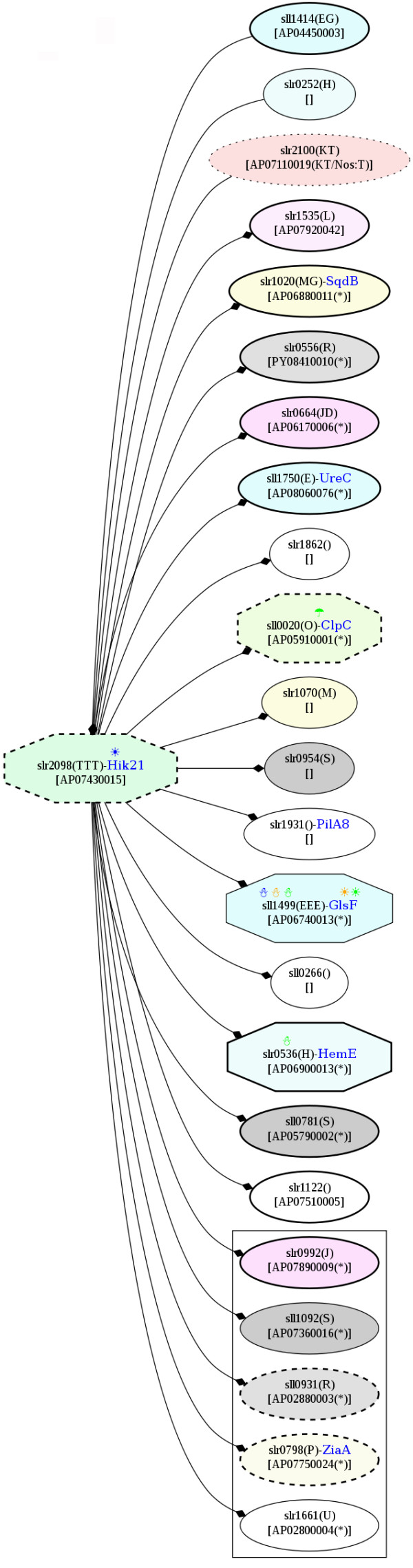
**Snapshots of AP07430015, Hik21, in the potential protein-protein interaction network in *Spirulina *in response to the temperature stresses**. The networks were constructed based on the data from Cyanobase.

Furthermore, the photosynthetic proteins that responded to the low-temperature stress are also associated with other cell processes. For example, the potential PPI indicated that HemE was associated with the cut-vertex two-component system, AP07430015 (slr2098 or Hik21) (Figure 15). ChlI has been reported to be involved in several processes, including the following: (i) protoporphyrin biosynthesis, which is an intermediate in chlorophyll biosynthesis [13], (ii) the signaling of the plastid to the nucleus [14], and (iii) regulation by thioredoxin [15].

Photosynthesis, which has been suggested to be maintained in cyanobacteria during low-temperature stress [4,16,17], has been shown to relate to many cell processes, including the oxidative stress response, nitrogen and ammonia assimilation, and the regulation of stress-related proteins. For example, SAH hydrolase, a common temperature stress protein, is not only involved in oxidative stress, but is also associated with photosynthesis during the methyl-group donation process [18]. Moreover, the association with nitrogen and ammonia assimilation and with stress-related protein regulation occurs via RpoD [19] and NADH dehydrogenase, respectively [20]. Su et al. reported that nitrogen and ammonia assimilation is tightly coordinated with photosynthesis [21], and Sakamoto and Bryant reported that low temperature causes nitrogen limitation [22]. Accordingly, the proteomic analyses of the response of *Spirulina *to low-temperature stress using the two approaches indicated that many proteins involved in nitrogen and ammonia assimilation were differentially expressed [4].

Another two up-regulated photosynthetic proteins, Rubisco and CcmM, were associated via CcmN and glycosyl transferase (Glpx), which was also detected as up-regulated protein under the low-temperature condition [Additional file 6 Table S4a]. However, the GlpX that was detected as an up-regulated protein in the present study was not the one encoded by the gene (AP07710045 or slr1125) shown in the potential PPI [Additional file 10 Figure S1b]. The Rubisco small subunit was found to be up-regulated in a moss, *Physcomitrella patens*, in response to abscisic acid [23]. Furthermore, Rubisco was interacted with a signaling protein, Hik23, in the PPI network. Recently, Pena et al reported that CcmM, CcmN and CcmA physically interact with Rubisco in the β-carboxysome of a cyanobacteria, *Methanosarcina thermophila*, to maintain its oxidizing interior by preventing the entry of thioredoxin and other endogenous reducing agents [24]. This data suggests that in response to low-temperature stress, a carbon dioxide concentrating mechanism was also involved, which might involve the signaling of Hik23 via the Rubisco small subunit.

### High-temperature responsive proteins and their potential PPI

**Figure 16:**
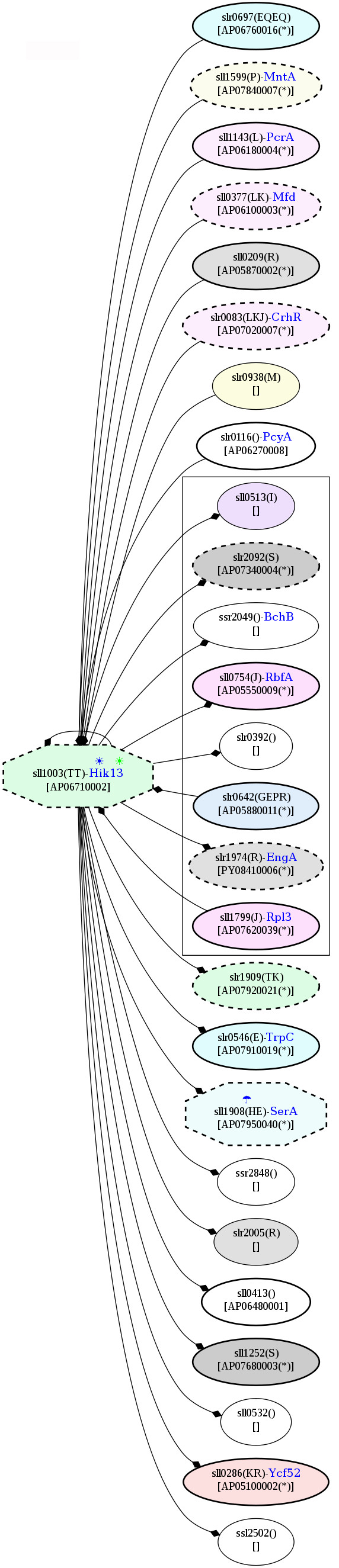
**Snapshots of AP06710002, Hik13, in the potential protein-protein interaction network in *Spirulina *in response to the temperature stresses**. The networks were constructed based on the data from Cyanobase.

PPI revealed that a high-temperature responsive signaling protein, AP06710002 or Hik13 interacts with the NarL-family response regulator, which involves in nitrogen and ammonia assimilation (Figure 16). Although the NarL-family response regulator was not detected in our study, the elevated level of Hik13 also highlights the association of temperature stress and nitrogen and ammonia assimilation. In addition, an up-regulated common temperature stress protein, DEAD/DEAH box helicase, and a down-regulated cold-responsive protein, D-3-phosphoglycerate dehydrogenase (SerA), which were identified in our study [Additional file 7 Table S4b], also showed potential interactions with Hik13 (Figure 16).

**Figure 17:**
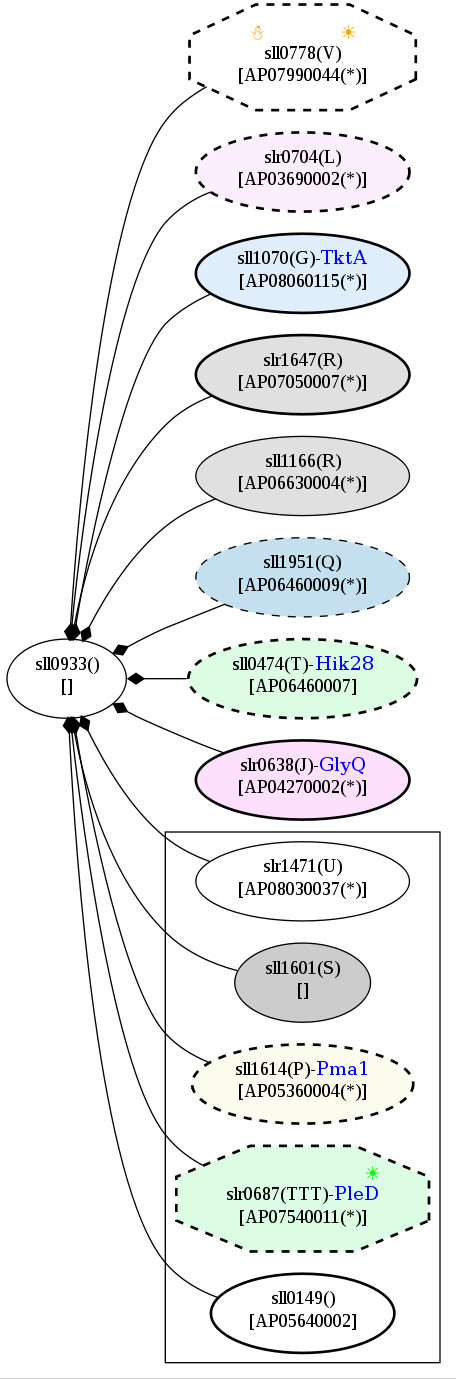
**Snapshots of AP06460007, Hik28, in the potential protein-protein interaction network in *Spirulina *in response to the temperature stresses**. The networks were constructed based on the data from Cyanobase.

**Figure 18:**
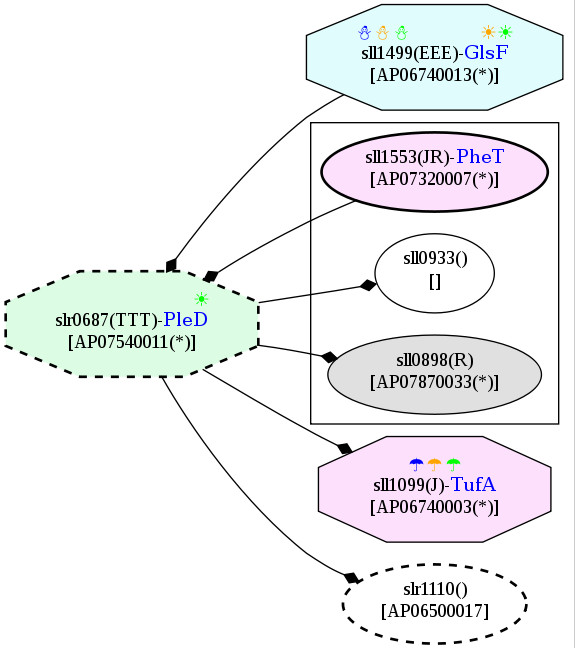
**Snapshots of AP07540011, response regulator PleD, in the potential protein-protein interaction network in *Spirulina *in response to the temperature stresses**. The networks were constructed based on the data from Cyanobase.

**Figure 19:**
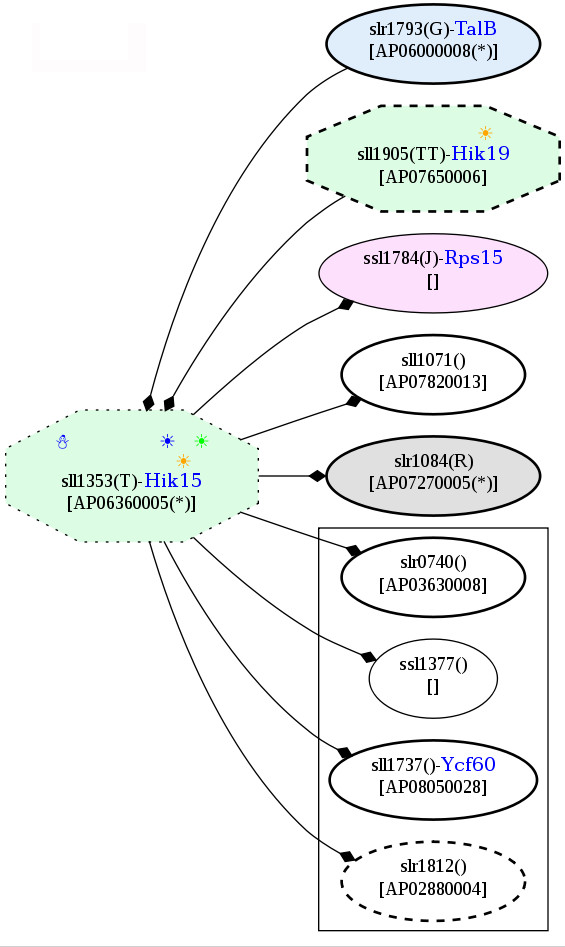
**Snapshots of AP06360005, Hik15, in the potential protein-protein interaction network in *Spirulina *in response to the temperature stresses**. The networks were constructed based on the data from Cyanobase.

According to the potential PPI network, another heat-responsive signaling protein, AP06460007 or Hik28, was revealed to have indirect association with AP07540011 or slr0687 or PleD, a down-regulated protein detected under the heat stress condition only (Figure 17). PleD was found to interact with elongation factor EfTu and GltB or GlsF, which are classified as cold-responsive and common temperature stress proteins, respectively (Figure 18). Moreover, a pair of signaling proteins, AP06360005 or Hik15 and AP07650006 or Hik19, represents direct associations in the PPI network (Figure 19). These interactions among proteins indicate the cross-talk of proteins under temperature stress.

### Proteins in common between the two temperatures and their potential PPI

**Figure 20:**
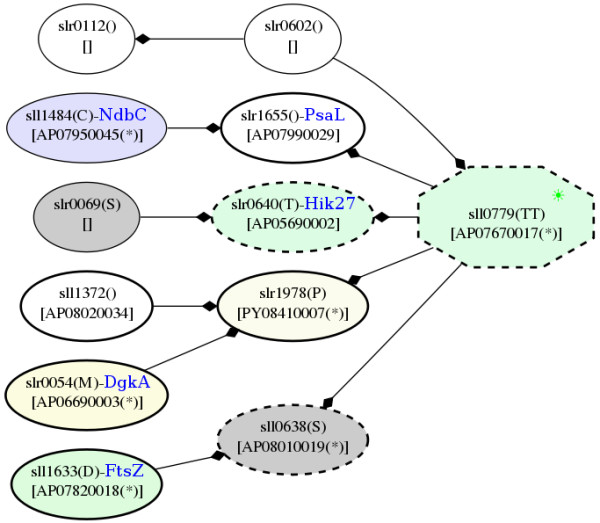
**Snapshots of AP07670017, two component sensor histidine kinase, in the potential protein-protein interaction network in *Spirulina *in response to the temperature stresses**. The networks were constructed based on the data from Cyanobase.

According to the potential PPI network, a signaling protein, AP04840005 has no potential interaction, whereas AP07670017 interacts with a cold-responsive protein, NADH dehydrogenase, as well as others, e.g., the cell division proteins (FtsZ) and PsaL (Figure 20). As mentioned above, Ndh was detected as an up-regulated cold-responsive protein; however, this NADH dehydrogenase (NdbC) is not encoded by the same gene as the previously discussed protein. Moreover, it is noteworthy that GltB interacted with the cut-vertex protein, two component system AP07430015 (Hik21), in the potential PPI network (Figure 15).

In term of transcriptional analysis, it should be highlighted that the regulation of common temperature stress proteins were regulated at the transcriptional level.

### PPI analysis by using yeast two hybrid system (Y2H)

The evidence of physical protein-protein interaction obtained by using Y2H suggests that the potential PPI network constructed by using bioinformatics approach gives a quite reliable prediction for the analysis of specificity and cross-talk of signaling components.

## Conclusion

According to the data obtained in our studies, in addition to of signal transduction systems, chaperones and DNA damage/DNA repairing proteins, nitrogen and ammonia assimilation is the common mechanism found after low- and high-temperature stress exposure in *Spirulina*. This result shows the linkage of temperature stress with nitrogen and ammonia assimilation. The results also indicate that low-temperature stress is tightly linked with oxidative stress and photosynthesis, as has been reported by several research groups, whereas no specific mechanism is revealed in the case of the high-temperature stress response. The differences in the response mechanisms of the cells to the two abiotic stress conditions are expected because of the different effects the two conditions have on the cells. Temperature reduction leads to impaired protein biosynthesis, stabilization of DNA and RNA secondary structures, and, particularly, to a reduction in membrane fluidity [25], whereas high-temperature stresses are known to cause protein aggregation and denaturation.

**Figure 21:**
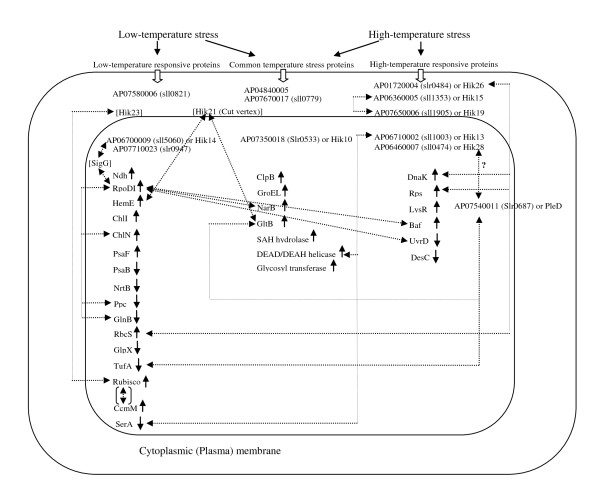
**Schematic diagram showing cross-talk among components found under the two temperature stress conditions**. All the proteins shown in this figure were identified as differentially expressed proteins in our studies, except the proteins with square parenthesis. The solid one-sided arrows represent up- or down-regulations, whereas the dashed two-sided arrows represent potential interactions obtained from the *Spirulina *PPI network. The dashed two-sided arrow in square parenthesis represents an interaction reported in the literature.

In the previous study, we reported evidence of cross-talk among three subcellular fractions, the plasma membrane, soluble and thylakoid membrane protein fractions, by considering each stress in isolation from other stresses [4,5]. However, in the present study, when the potential PPI network is analyzed in coordination with the differentially expressed proteins that were identified in each temperature stress condition (Figure 21), the cross-talk of signaling pathways are observed. As shown in Figure 21, the two temperature stresses share common elements that are potential points of cross-talk. For example, Hik14 indirectly interacted with RpoDI or SigA, and RpoDI shows potential interactions with several differentially expressed proteins identified under low-temperature, high-temperature and both conditions. Further evidence of common elements is the detection of a certain group of proteins under many types of stress, e.g., proteins related to oxidative stress were identified under oxidative stress [26], low-temperature stress [4], salt stress and hyperosmotic stress [27,28]. A possible reason for this might be the requirement of the same protective action or, at least, some common elements under these stress conditions. Moreover, in this study, we found three signaling proteins (two in the plasma membrane and one in the soluble fraction) that were triggered by the two different temperature stresses (Figure 21), supporting the common element sharing hypothesis mentioned above.

In spite of overlapping response mechanisms, a specific response was also observed in case of the low-temperature stress exposure. We identified many differentially expressed photosynthetic related proteins under this particular condition. Induction of the appropriate response that would be suited to the stress condition and avoidance of the high energy cost might be the reasons for this specific response.

In the present study, protein-protein interactions, which are well known to be tightly link with signal transduction cascades [29], were constructed by mapping the differentially expressed proteins of *Spirulina *under two types of stress, using two proteomic approaches, onto the PPI network of *Synechocystis*, which has been constructed using the results obtained from Y2H analyses of *Synechocystis*. The Y2H data obtained in this study suggest that the potential PPI network gives quite reliable potential interactions for *Spirulina*. Where proteome analyses of knockout mutants have not been carried out to directly examine for specificity or cross-talk of signaling components due to the lack of a stable transformation system in *Spirulina*, the bioinformatics approach employed in this study helps in analysis of phenomena.

## Declaration of competing interests

The authors declare that they have no competing interests.

## Authors' contributions

PK carried out the transcriptional analysis by RT-PCR and participated in the protein-protein interaction studies by yeast two hybrid system. JS carried out the protein clustering and the protein-protein interaction network construction. MS carried out the protein analysis by using liquid chromatography-tandem mass spectrometry and participated in the file conversion from (*.wiff) to *.mzXML and then to *.mgf files. RY carried out the culturing of *Spirulina *and membrane preparation. PP participated in the protein-protein interaction studies by yeast two hybrid system. WJ participated in the DNA-probe design for transcriptional analysis. SR participated in the file conversion from (*.wiff) to *.mzXML and then to *.mgf files. SC participated in the providing of genome database used for protein identification. AH conceived of the study, carried out the data interpretation and its design and coordination. All authors read and approved the final manuscript.

## Supplementary Material

Additional file 1 **Table S1a**. A result file of soluble fraction obtained by using ProteinPilot™software. Click here for file

Additional file 2 **Table S1b**. A result file of plasma membrane fraction obtained by using ProteinPilot™software. Click here for file

Additional file 3 **Table S1c**. A result file of thylakoid membrane fraction obtained by using ProteinPilot™software. Click here for file

Additional file 4 **Table S2**. Details on primers and conditions used for transcriptional analysis by RT-PCR. Click here for file

Additional file 5 **Table S3**. List of differentially expressed proteins (after having been filtering by statistical criteria mentioned in Material & Methods) detected by the two techniques under low-temperature and high temperature stresses. The column names, coldLC, cold2D and heat2D, represent the conditions and techniques, whereas the fractions where the proteins were detected are listed by abbreviation S, P and T for soluble, plasma membrane and thylakoid membrane fractions, respectively. Click here for file

Additional file 6 **Table S4a**. List of differentially expressed proteins (after having been filtering by statistical criteria mentioned in Material & Methods) detected by the two techniques under low-temperature stress only. The column names, coldLC, cold2D and heat2D, represent the conditions and techniques, whereas the fractions where the proteins were detected are listed by abbreviation S, P and T for soluble, plasma membrane and thylakoid membrane fractions, respectively. Click here for file

Additional file 7 **Table S4b**. List of differentially expressed proteins (after having been filtering by statistical criteria mentioned in Material & Methods) under high-temperature stress only. The column names, coldLC, cold2D and heat2D, represent the conditions and techniques, whereas the fractions where the proteins were detected are listed by abbreviation S, P and T for soluble, plasma membrane and thylakoid membrane fractions, respectively. Click here for file

Additional file 8 **Table S4c**. List of differentially expressed proteins (after having been filtering by statistical criteria mentioned in Material & Methods) detected by the two techniques under both temperature stresses. The column names, coldLC, cold2D and heat2D, represent the conditions and techniques, whereas the fractions where the proteins were detected are listed by abbreviation S, P and T for soluble, plasma membrane and thylakoid membrane fractions, respectively. Click here for file

Additional file 9 **Figure S1a**. Potential protein-protein interaction (PPI) network under the two temperature stress conditions of the three subcellular fractions. (a) PPI of soluble fraction in response to low-temperature stress analyzed by 2D-DIGE and LC-MS/MS. Click here for file

Additional file 10 **Figure S1b**. Potential protein-protein interaction (PPI) network under the two temperature stress conditions of the three subcellular fractions. (b) PPI of soluble fraction in response to low-temperature stress analyzed by LC-MS/MS. Click here for file

Additional file 11 **Figure S1c**. Potential protein-protein interaction (PPI) network under the two temperature stress conditions of the three subcellular fractions. (c) PPI of plasma membrane fraction in response to low-temperature stress analyzed by 2D-DIGE and LC-MS/MS. Click here for file

Additional file 12 **Figure S1d**. Potential protein-protein interaction (PPI) network under the two temperature stress conditions of the three subcellular fractions. (d) PPI of plasma membrane fraction in response to low-temperature stress analyzed by LC-MS/MS. Click here for file

Additional file 13 **Figure S1e**. Potential protein-protein interaction (PPI) network under the two temperature stress conditions of the three subcellular fractions. (e) PPI of thylakoid membrane fraction in response to low-temperature stress analyzed by 2D-DIGE and LC-MS/MS. Click here for file

Additional file 14 **Figure S1f**. Potential protein-protein interaction (PPI) network under the two temperature stress conditions of the three subcellular fractions. (f) PPI of thylakoid membrane fraction in response to low-temperature stress analyzed by LC-MS/MS. Click here for file

Additional file 15 **Figure S1g**. Potential protein-protein interaction (PPI) network under the two temperature stress conditions of the three subcellular fractions. (g) PPI of soluble fraction in response to high-temperature stress analyzed by 2D-DIGE. Click here for file

Additional file 16 **Figure S1h**. Potential protein-protein interaction (PPI) network under the two temperature stress conditions of the three subcellular fractions. (h) PPI of plasma membrane fraction in response to high-temperature stress analyzed by 2D-DIGE. Click here for file

Additional file 17 **Figure S1i**. Potential protein-protein interaction (PPI) network under the two temperature stress conditions of the three subcellular fractions. (i) PPI of thylakoid membrane fraction in response to high-temperature stress analyzed by 2D-DIGE. Click here for file

Additional file 18 **Figure S2**. Transcriptional analyses of some differentially expressed proteins using RT-PCR (top panel) and protein expression levels (bottom panel) of the designated open-reading frames subjected to the transcriptional analysis. (Note: In case of proteins detected by LC-MS/MS technique, the protein expression levels at 45, 90 and 180 min compared to that of at 0 min are 115:114, 116:114 and 117:114 ratios of iTRAQ, respectively. In case of proteins detected by 2D-DIGE, the protein expression levels at 0, 45, 90 and 180 min are log-value of the protein-spot density at the designated time period compared to that of at 0 min.). Click here for file

Additional file 19 **Table S5**. List of differentially expressed proteins (after having been filtering by statistical criteria mentioned in Material & Methods) detected by LC-MS/MS technique under low-temperature stress only. The fractions where the proteins were detected are listed by abbreviation S, P and T for soluble, plasma membrane and thylakoid membrane fractions, respectively, whereas the column names, Cluster no._S, Cluster no._P and Cluster no._T represent the protein-expression-pattern cluster number of the designated protein found in soluble, plasma membrane and thylakoid membrane fractions, respectively. Click here for file
