# Supplementary material for: Comparative analysis of the Spirulina platensis subcellular proteome in response to low- and high-temperature stresses: uncovering cross-talk of signaling components
Source: Proteome Sci. 2011 Jul 15;9:39. doi: 10.1186/1477-5956-9-39 (PMC3162491; doi:10.1186/1477-5956-9-39)
Supplement: Additional file 4 — Table S2. Details on primers and conditions used for transcriptional analysis by RT-PCR. [file 1477-5956-9-39-S4.DOC]

| Orf  number | Primer name | Oligonucleotide primers  5′ 3′ | PCR Condition |  |
| --- | --- | --- | --- | --- |
| AP08030057 | AP08030057F  AP08030057R | GTGTCATCTAACGTCACTGCAGGAGC  TCAGCCTGTGTTACGCATTCCG | 95°C 3 min.  95°C 45 sec.  49°C 1 min.  72°C 4 min | 35 cycles  (PCR amplification) |
|  |  |  | 72°C 10min | 1 cycle (final extension) |
| AP05380002 | AP05380002F  AP05380002R | CAGTGGCGGCGAAAC  ATATCTTTT  TGCCAGCTTGTCCGGG  TCTT | 94°C 2 min 94°C 45 sec  52°C 30 sec  72°C 1 min | 35 cycles  (PCR amplification) |
|  |  |  | 72°C 10 min | 1cycle (final extension) |
| AP06740013 | AP06740013F    AP06740013R | GGACGACTTCTCGGAT  TGGAGG  GCAGTAACTTCGCCAA  TTCC | 94°C 2 min 94°C 45 sec  52°C 30 sec  72°C 1 min | 35 cycles  (PCR amplification) |
|  |  |  | 72°C 10 min | 1 cycle (final extension) |
| AP06900013 | AP06900013F  AP06900013R | ATGACCGACTCGACCC  AAACC  TTAGACATGAGTTGCC  AATAGTTGATCTG | 94°C 2 min  94°C 45 sec  50°C 30 sec  72°C 1.30 min | 35 cycles  (PCR amplification) |
|  |  |  | 72°C 10 min | 1 cycle (final extension) |
| AP07580006 | AP07580006F  AP07580006R | ATGAGTTATAAACAGT  CAAA  TTACCATGTTAGATTT  CC | 94°C 2 min 94°C 45 sec  52°C 30 sec  72°C 2 min | 35 cycles  (PCR amplification) |
|  |  |  | 72°C 10 min | 1 cycle (final extension) |
| AP07670017 | AP07670017F  AP07670017R | ATGATTGCCAATATTAGGTTCC  TCAACTCAGGGTAGCTTAATC | 95°C 3 min  95°C 45 sec  51°C 1 min  72°C 3.30 min | 35 cycles  (PCR amplification) |
|  |  |  | 72°C 10min | 1 cycle (final extension) |
| AP08030020 | AP08030020F  AP08030020R | ATGACCGTACTTGAAC  AGGG  TTACATGGCCCGTTCTG | 94°C 2 min  94°C 30 sec  51°C 30 sec  68°C 2 min | 35 cycles  (PCR amplification) |
| AP08040017 | AP08040017F  AP08040017R | ATGGCGAAAATCGTAG  CC  TTACATCATTCCCATGCCAC | 94°C 2 min  94°C 45 sec  52°C 30 sec  72°C 2 min | 35 cycles  (PCR amplification) |
|  |  |  | 72°C 10 min | 1 cycle (final extension) |
