# Supplementary figures and images for: Comparative analysis of the Spirulina platensis subcellular proteome in response to low- and high-temperature stresses: uncovering cross-talk of signaling components
Source: Proteome Sci. 2011 Jul 15;9:39. doi: 10.1186/1477-5956-9-39 (PMC3162491; doi:10.1186/1477-5956-9-39)

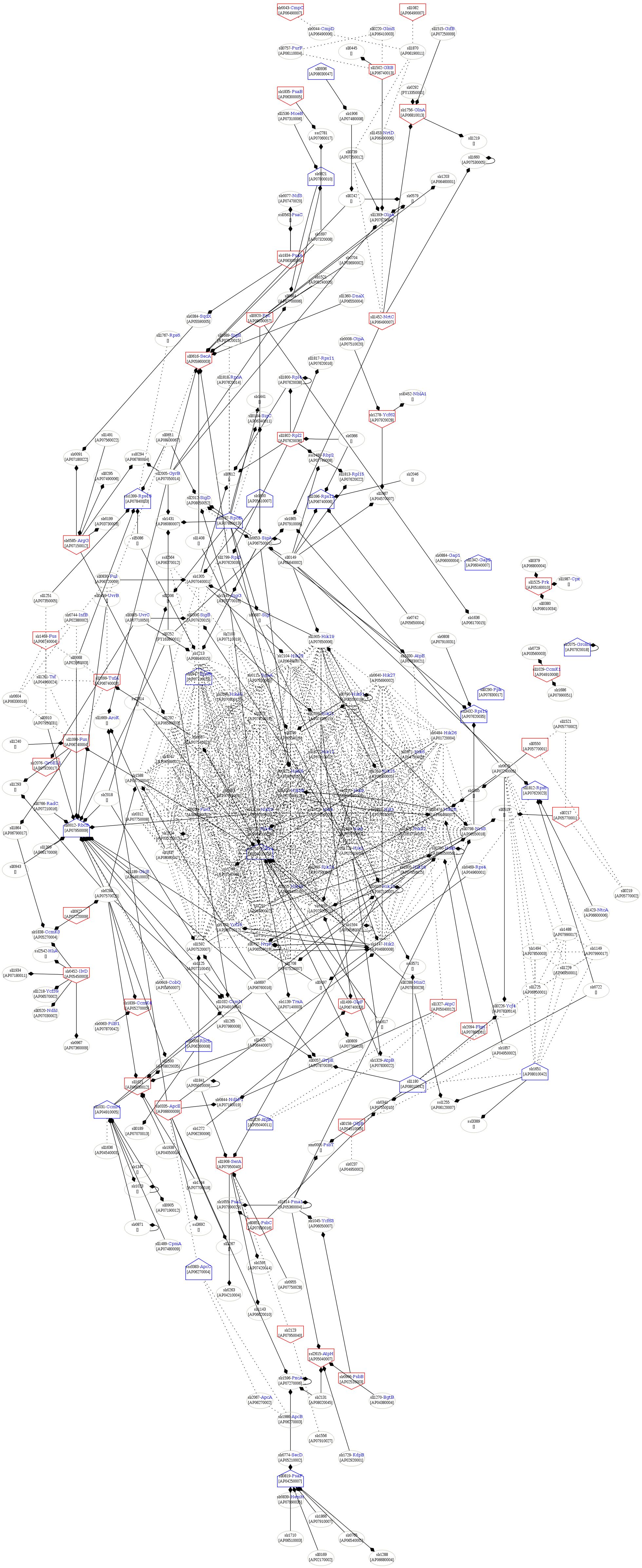

Supplement: Additional file 9 — Figure S1a. Potential protein-protein interaction (PPI) network under the two temperature stress conditions of the three subcellular fractions. (a) PPI of soluble fraction in response to low-temperature stress analyzed by 2D-DIGE and LC-MS/MS. [file 1477-5956-9-39-S9.PNG]

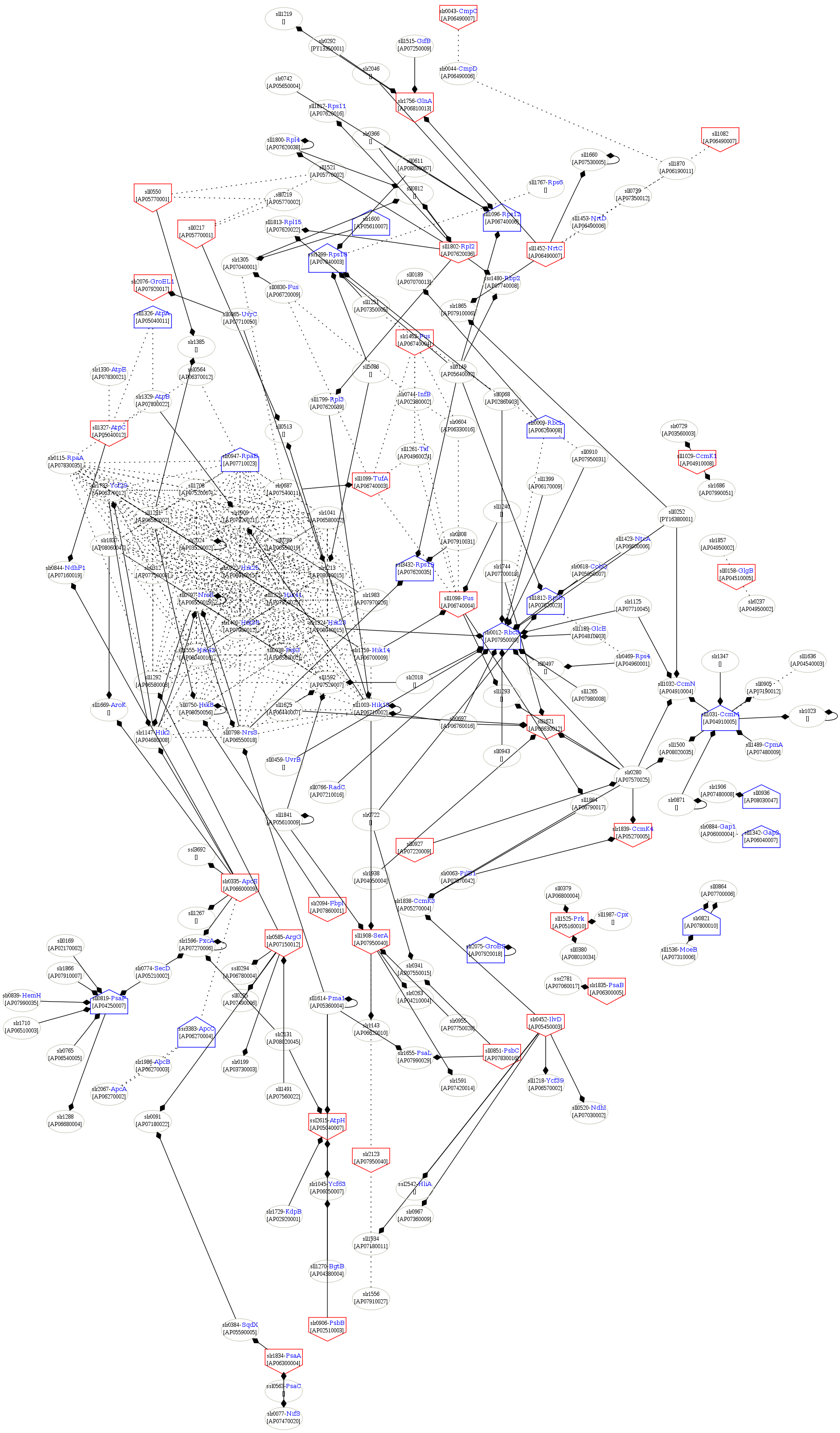

Supplement: Additional file 10 — Figure S1b. Potential protein-protein interaction (PPI) network under the two temperature stress conditions of the three subcellular fractions. (b) PPI of soluble fraction in response to low-temperature stress analyzed by LC-MS/MS. [file 1477-5956-9-39-S10.PNG]

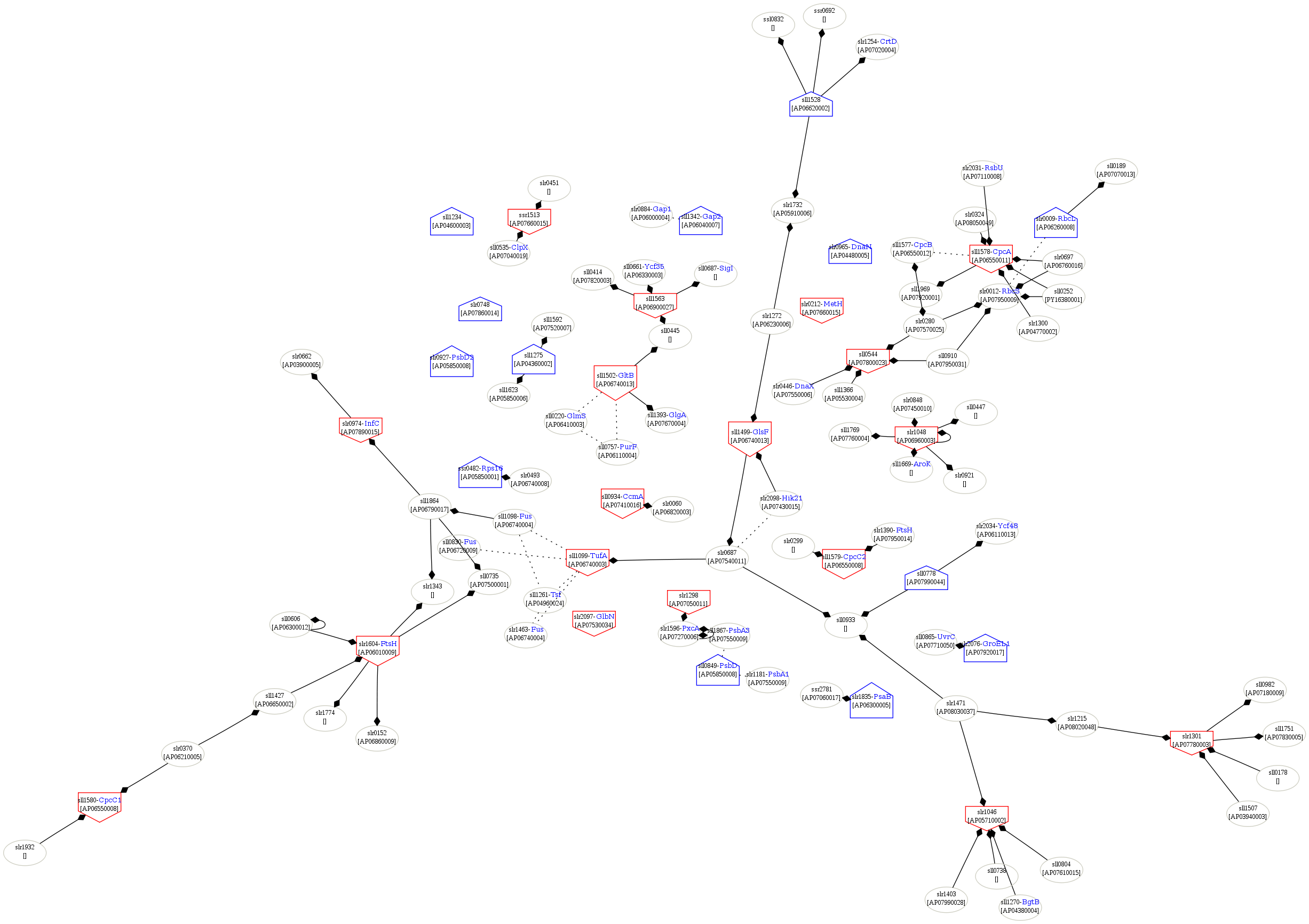

Supplement: Additional file 11 — Figure S1c. Potential protein-protein interaction (PPI) network under the two temperature stress conditions of the three subcellular fractions. (c) PPI of plasma membrane fraction in response to low-temperature stress analyzed by 2D-DIGE and LC-MS/MS. [file 1477-5956-9-39-S11.PNG]

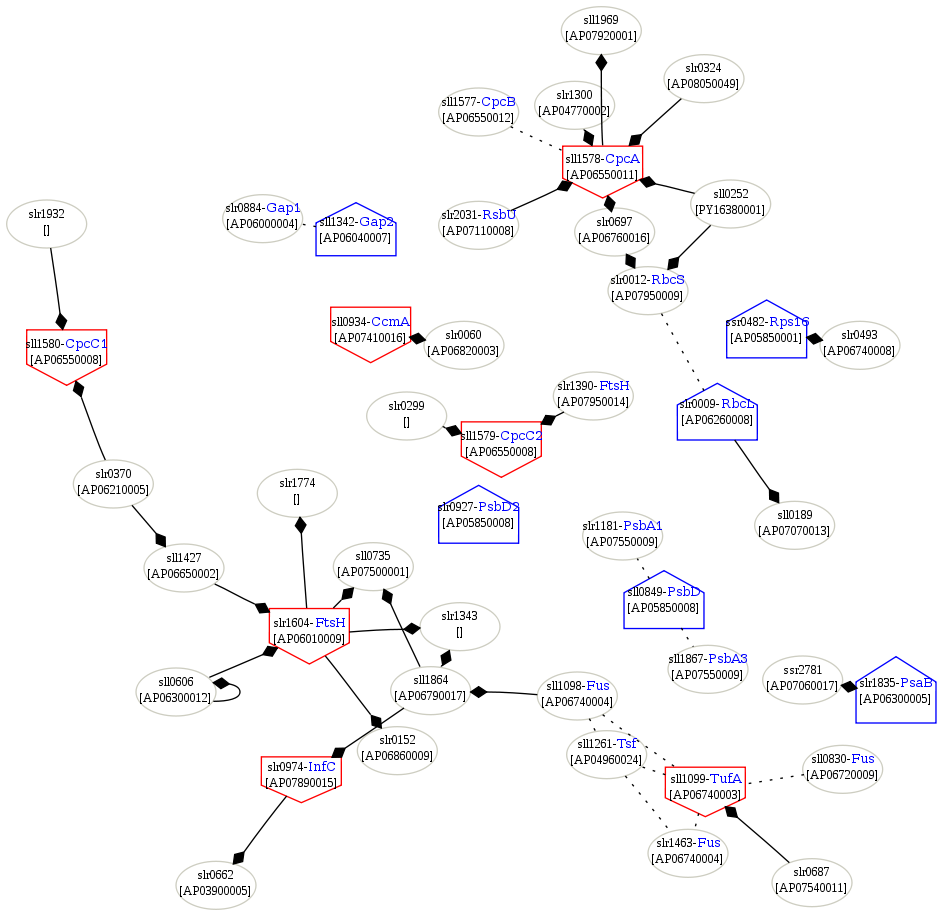

Supplement: Additional file 12 — Figure S1d. Potential protein-protein interaction (PPI) network under the two temperature stress conditions of the three subcellular fractions. (d) PPI of plasma membrane fraction in response to low-temperature stress analyzed by LC-MS/MS. [file 1477-5956-9-39-S12.PNG]

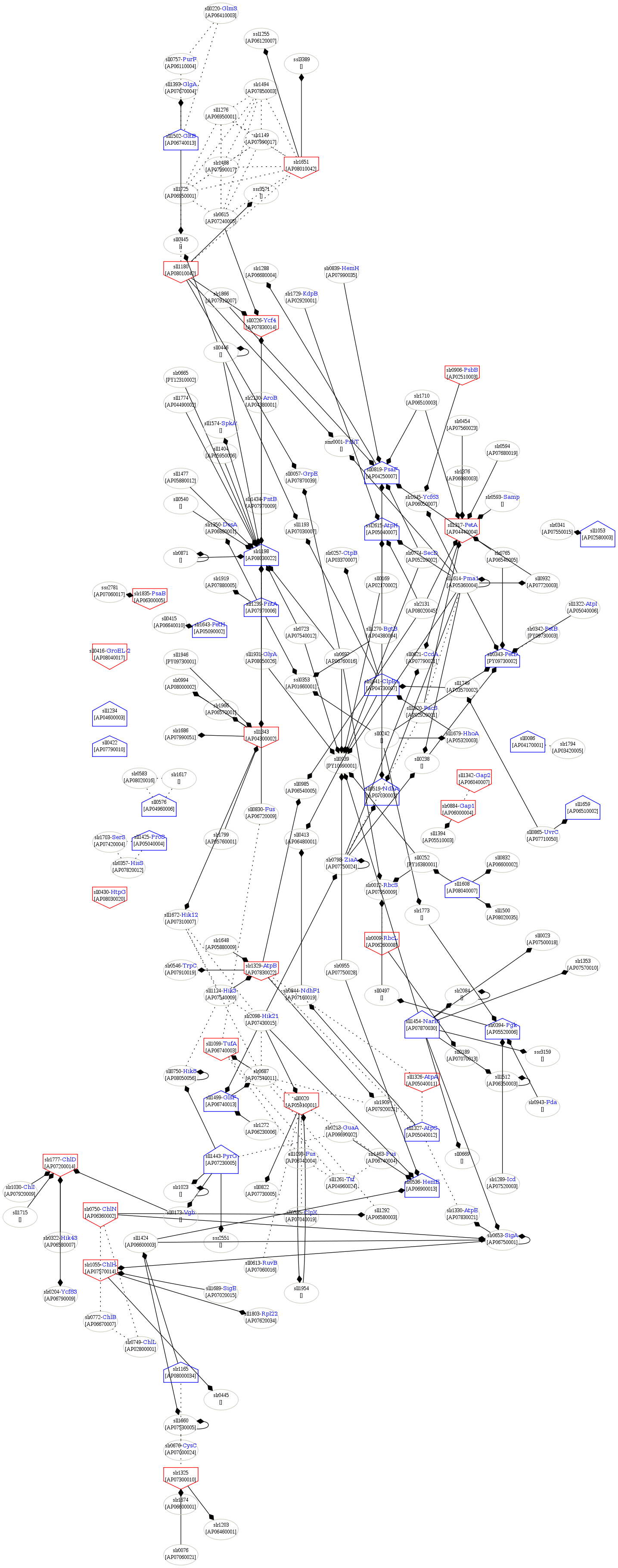

Supplement: Additional file 13 — Figure S1e. Potential protein-protein interaction (PPI) network under the two temperature stress conditions of the three subcellular fractions. (e) PPI of thylakoid membrane fraction in response to low-temperature stress analyzed by 2D-DIGE and LC-MS/MS. [file 1477-5956-9-39-S13.PNG]

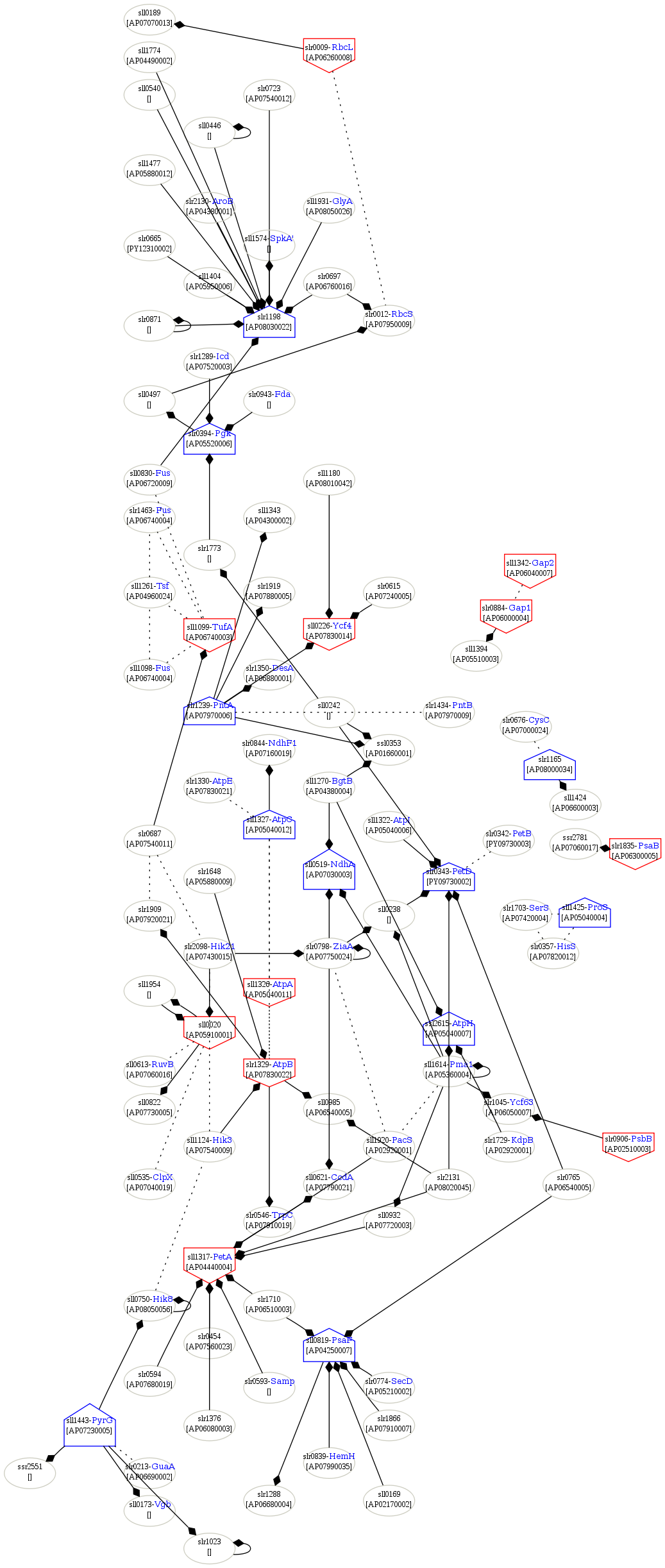

Supplement: Additional file 14 — Figure S1f. Potential protein-protein interaction (PPI) network under the two temperature stress conditions of the three subcellular fractions. (f) PPI of thylakoid membrane fraction in response to low-temperature stress analyzed by LC-MS/MS. [file 1477-5956-9-39-S14.PNG]

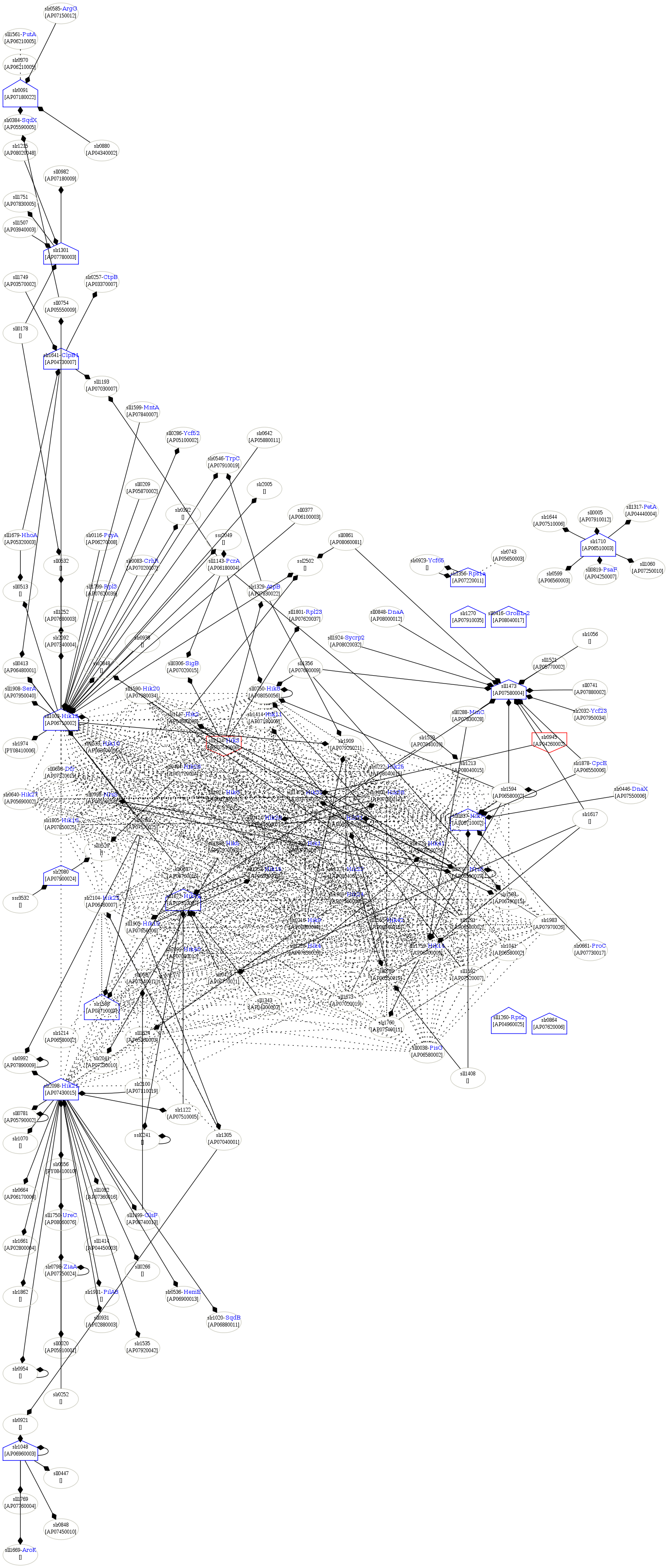

Supplement: Additional file 15 — Figure S1g. Potential protein-protein interaction (PPI) network under the two temperature stress conditions of the three subcellular fractions. (g) PPI of soluble fraction in response to high-temperature stress analyzed by 2D-DIGE. [file 1477-5956-9-39-S15.PNG]

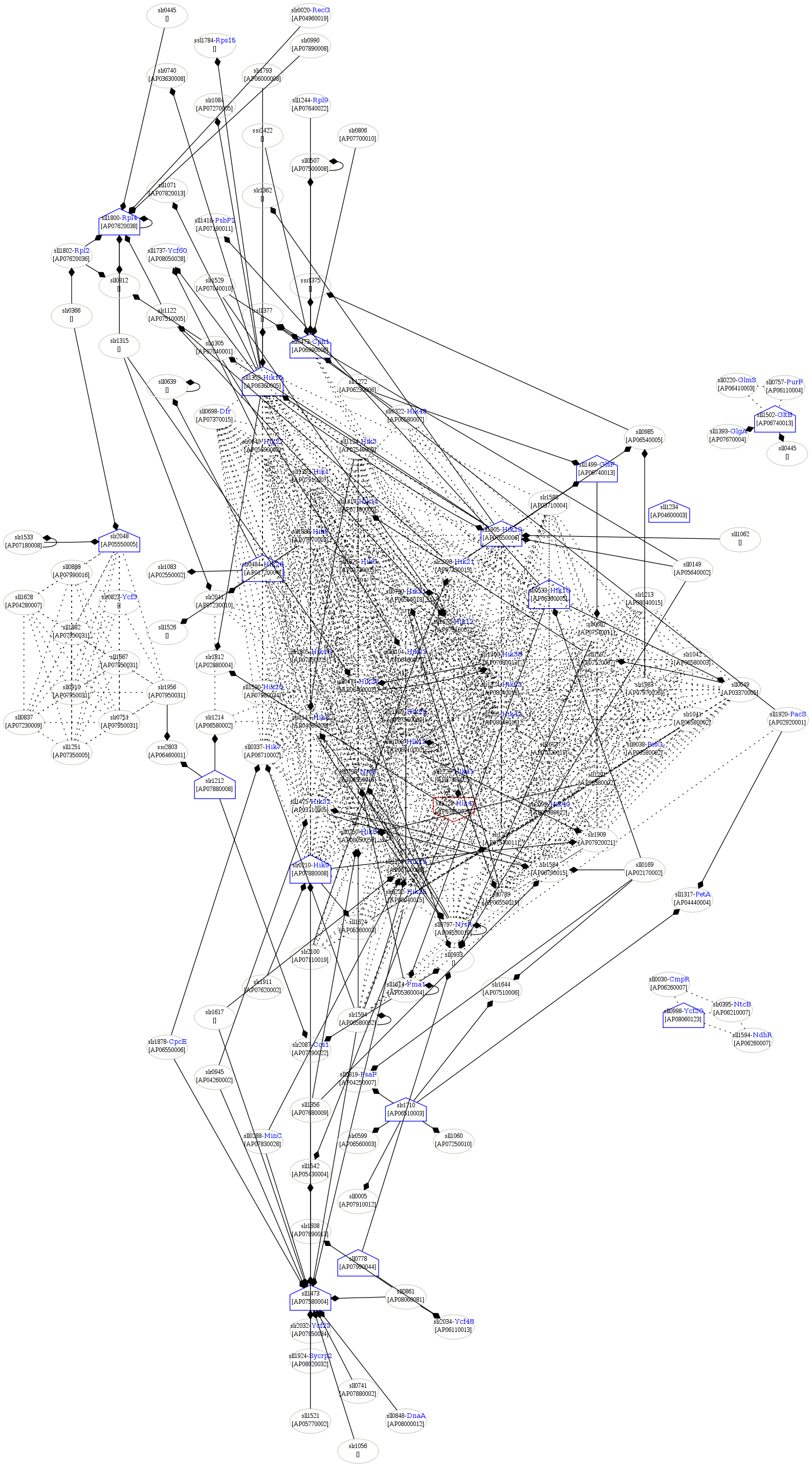

Supplement: Additional file 16 — Figure S1h. Potential protein-protein interaction (PPI) network under the two temperature stress conditions of the three subcellular fractions. (h) PPI of plasma membrane fraction in response to high-temperature stress analyzed by 2D-DIGE. [file 1477-5956-9-39-S16.PNG]

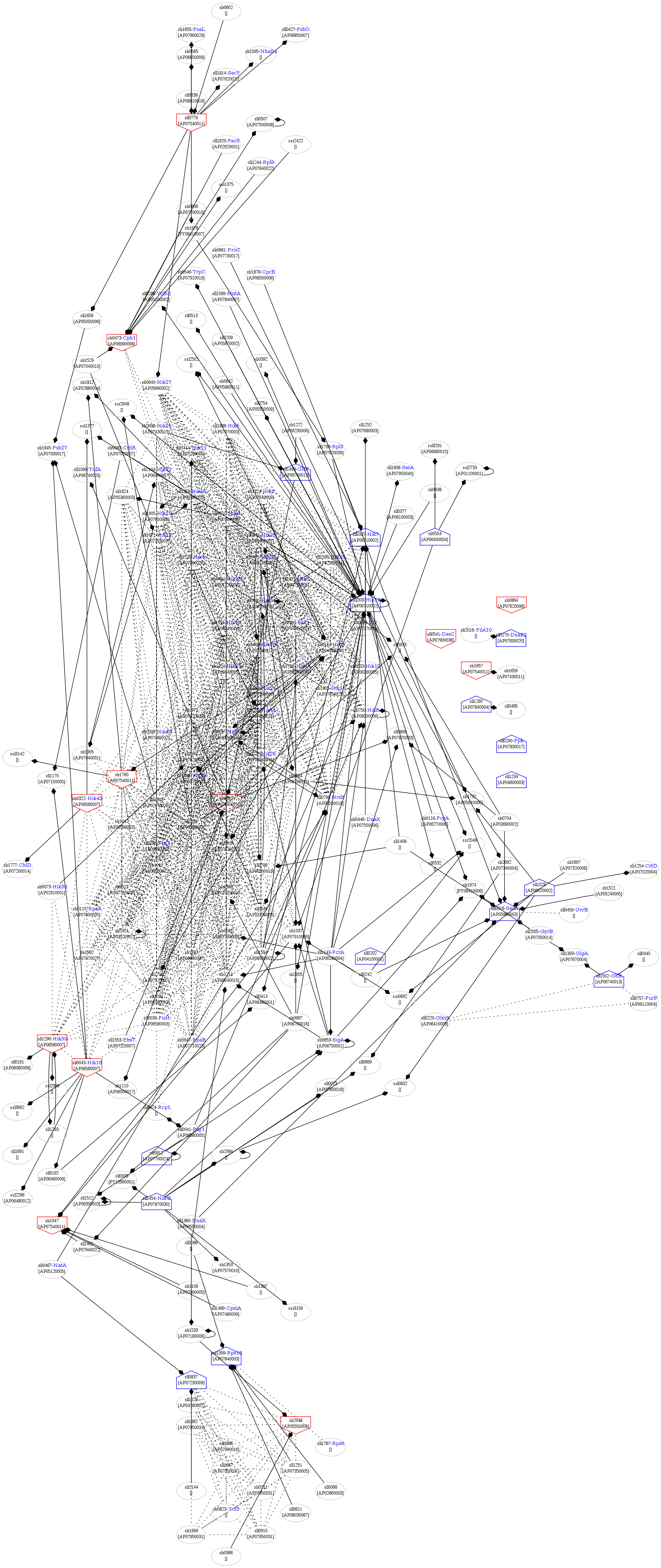

Supplement: Additional file 17 — Figure S1i. Potential protein-protein interaction (PPI) network under the two temperature stress conditions of the three subcellular fractions. (i) PPI of thylakoid membrane fraction in response to high-temperature stress analyzed by 2D-DIGE. [file 1477-5956-9-39-S17.PNG]
