# Supplementary material for: Comparative analysis of the Spirulina platensis subcellular proteome in response to low- and high-temperature stresses: uncovering cross-talk of signaling components
Source: Proteome Sci. 2011 Jul 15;9:39. doi: 10.1186/1477-5956-9-39 (PMC3162491; doi:10.1186/1477-5956-9-39)
Supplement: Additional file 18 — Figure S2. Transcriptional analyses of some differentially expressed proteins using RT-PCR (top panel) and protein expression levels (bottom panel) of the designated open-reading frames subjected to the transcriptional analysis. (Note: In case of proteins detected by LC-MS/MS technique, the protein expression levels at 45, 90 and 180 min compared to that of at 0 min are 115:114, 116:114 and 117:114 ratios of iTRAQ, respectively. In case of proteins detected by 2D-DIGE, the protein expression levels at 0, 45, 90 and 180 min are log-value of the protein-spot density at the designated time period compared to that of at 0 min.). [file 1477-5956-9-39-S18.DOC]

AP05380002 DEAD/DEAH box helicase domain protein (membrane helicase)


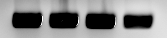


**AP05380002**

**(1263bp)**

**16SrRNA**

**(464bp)**


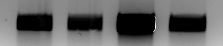


Spot# 2657 (SOL): DEAD/DEAH box helicase domain protein (membrane helicase)

AP06740013 Ferredoxin-glutamate synthase


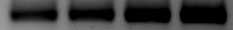

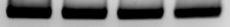


**AP06740013**

**(176bp)**

**16S rRNA**

**(464bp)**

Spot# 1266 (SOL): Ferredoxin-glutamate synthase

AP06900013 Uroporphyrinogen decarboxylase


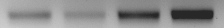

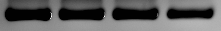


**AP06900013**

**(1065bp)**

**16S rRNA**

**(464bp)**

Spot# 3080 (TM): Uroporphyrinogen decarboxylase

AP07580006 Response regulator (CheY like-GGDEF containing protein)


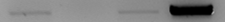

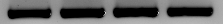


**AP07580006**

**(1797bp)**

**16S rRNA**

**(464bp)**

Spot# 2310 (PM): Response regulator (CheY like-GGDEF containing protein)

AP07670017 Two-component sensor histidine kinase


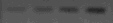

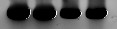


**AP07670017**

**(3246bp)**

**16S rRNA**

**(464bp)**

Spot# 3486 (PM): Two-component sensor histidine kinase

AP08030020 Heat shock protein 90


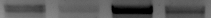

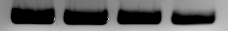


**AP08030020**

**(1986bp)**

**16S rRNA**

**(464bp)**

Spot# 1675 (TM): Hsp90

AP08030057 PEP carboxylase


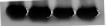


**AP08030057**

**(3063bp)**

**16S rRNA**

**(464bp)**


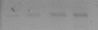


(SOL) Phosphoenolpyruvate carboxylase


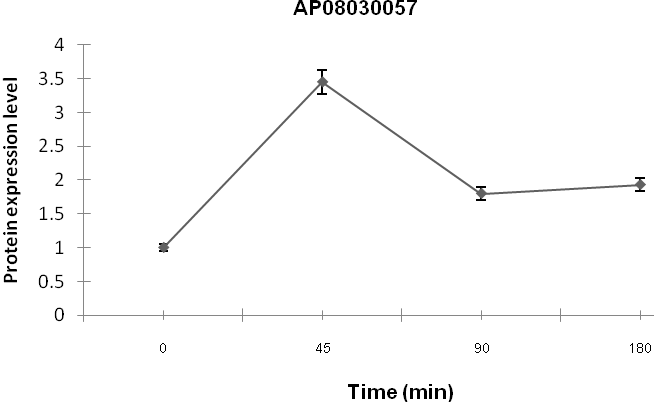


AP08040017 Chaperone GroEL


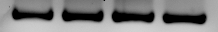

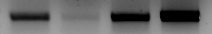


**AP08040017**

**(1677bp)**

**16S rRNA**

**(464bp)**

Spot# 2313 (TM): GroEL
